# Integrating network pharmacology, UPLC-Q–TOF–MS and molecular docking to investigate the effect and mechanism of Chuanxiong Renshen decoction against Alzheimer's disease

**DOI:** 10.1186/s13020-022-00698-1

**Published:** 2022-12-24

**Authors:** Zhuo Jun Shen, Yun Bo Fu, Jin Ling Hou, Lu Ning Lin, Xiao Yan Wang, Chang Yu Li, Yuan Xiao Yang

**Affiliations:** 1 School of Pharmacy, Hangzhou Medical College, Hangzhou, China; 2 Department of Pharmacy, Zhejiang Chinese Medical University, Hangzhou, China; 3 School of Basic Medical Sciences and Forensic Medicine, Hangzhou Medical College, Hangzhou, China

**Keywords:** Alzheimer's disease (AD)_1_, Chuanxiong Renshen decoction (CRD)_2_, Ultraperformance liquid chromatography quadrupole time-of-flight tandem mass spectrometry (UPLC-Q–TOF–MS)_3_, Network pharmacology_4_, Molecular docking_5_

## Abstract

**Background and aim:**

Chuanxiong Renshen decoction (CRD) is a traditional Chinese medicine compound used to treat Alzheimer's disease (AD). However, the effects and active ingredients of CRD and its mechanism have not been clarified. We aimed to determine the neuroprotective effects of CRD in a triple-transgenic mouse model of AD (3 × Tg-AD) and investigate the possible active ingredients and their mechanisms.

**Methods:**

Morris water maze (MWM) tests were used to determine the protective effect of CRD on learning and memory ability. Afterward, we used brain tissue staining, immunofluorescent staining and western blotting to detect the neuroprotective effects of CRD. Ultraperformance liquid-chromatography-quadrupole–time-of-flight tandem mass spectrometry (UPLC-Q–TOF–MS) was applied to determine the ingredients of CRD, and the potential AD targets were obtained from DisGeNET and the GeneCards database. The protein‒protein interaction (PPI) network was built with the additional use of STRING 11.0. Metascape was used in the pathway enrichment analysis. Discovery Studio 2016 (DS) software was used to analyze the binding ability of CRD and AD-related genes. Finally, we verified the regulatory effect of CRD on the predicted core targets *EGFR* and *CASP3* by western blotting.

**Results:**

Our study indicated that CRD can significantly improve learning and memory, reduce the expression of Aβ and protect neurons. A total of 95 ingredients were identified in the CRD. Then, 25 ingredients were identified in serum, and 5 ingredients were identified in the brain tissue homogenate. PPI network analysis identified *CASP3*, *EGFR*, *APP*, *CNR1*, *HIF1A*, *PTGS2* and *MTOR* as hub targets. KEGG and GO analyses revealed that the TNF signaling pathway and MAPK signaling pathway were enriched in multiple targets. The results of molecular docking proved that the binding of the ingredients with potential key targets was excellent. The western blotting results showed that CRD could significantly reduce the expression of *CASP3* and *EGFR* in the hippocampus of 3 × Tg-AD mice. Combined with literature analysis, we assumed the neuroprotective effect of CRD on AD may occur through regulation of the MAPK signaling pathway.

**Conclusion:**

CRD significantly alleviated injury in 3 × Tg-AD mice. The possible active ingredients are ferulic acid, rutin, ginsenoside Rg1 and panaxydol. The therapeutic effect of CRD on AD is achieved through the downregulation of *CASP3* and *EGFR*. The neuroprotective effect of CRD on AD may occur through regulation of the MAPK signaling pathway.

**Supplementary Information:**

The online version contains supplementary material available at 10.1186/s13020-022-00698-1.

## Background

AD is a neurodegenerative disease and has become the seventh leading cause of death in the world [1]. According to the World Health Organization, there are approximately 50 million people worldwide with dementia [2]. The total number of dementia patients is predicted to reach 82 million by 2030 and 152 million by 2050 [3]. The continuous course of AD imposes a heavy economic and psychological burden on society, families and individuals. Donepezil and other cholinesterase inhibitors are mainly used in clinical treatment, but the effect is not satisfactory [4, 5].

According to traditional Chinese medicine (TCM), the etiology of Alzheimer's disease is mainly caused by the deficiency of essence, qi and blood, the emptiness of the pulp sea, the internal obstruction of qi, fire, phlegm, and blood stasis, and the disturbance of clearing the orifice [6]. CRD is a TCM prescription for the treatment of AD, which is administered by some Chinese doctors in practice. The results of clinical application show that CRD has a good anti-AD effect, and there are no adverse reactions. CRD, which consists of *Conioselinum anthriscoides 'Chuanxiong' (Chuanxiong), Panax ginseng C.A. (Renshen), Pueraria montana var. lobata (Willd.) Maesen & S.M. Almeida ex Sanjappa & Predeep (Gegen), Ginkgo Biloba L. (Yinxingye),* and *Reynoutria multiflora (Thunb.) Moldenke (Heshouwu),* promotes qi and blood circulation. *Panax ginseng C.A.Mey. (Renshen)* is used as a primary medicine to replenish qi, strengthen the spleen, and improve intelligence and calm nerves. It is supplemented by *Conioselinum anthriscoides 'Chuanxiong' (Chuanxiong)* to promote blood circulation and eliminate blood stasis. Contemporary studies have shown that *Conioselinum anthriscoides 'Chuanxiong' (Chuanxiong)* can promote learning and memory [7]. *Panax ginseng C.A.Mey. (Renshen)* has a significant protective effect against learning and memory disorders in various experimental animals [8]. Moreover, some studies have shown that *Pueraria montana var. lobata (Willd.) Maesen & S.M. Almeida ex Sanjappa & Predeep (Gegen)* is effective in the treatment of aging and age-related diseases [9]. Standardized *Ginkgo Biloba L. (Yinxingye)* extract is a popular dietary supplement to improve memory and age-related cognitive loss [10]. *Reynoutria multiflora (Thunb.) Moldenke (Heshouwu)* can also improve learning and memory impairment in sporadic AD mice [11].

UPLC-Q–TOF–MS is a high-throughput analytical technique widely used in drug ingredient analysis. Li Xu et al. used UPLC-Q–TOF–MS to develop a rapid method for characterizing the chemical constituents in Gandou decoction [12]. Wei et al. applied UPLC-Q–TOF–MS to elucidate the mechanisms by which lignans in *S. chinensis* function in the treatment of AD [13]. We used this technique to analyze the ingredients of CRD. Network pharmacology is a theory based on systems biology, emphasizing the multichannel regulation of signals, which is consistent with the characteristics of multi-ingredient and multitarget TCM [14]. Zhang et al. analyzed the treatment of rosacea and AD through network pharmacology [15]. Network pharmacology has also played an important role in elucidating the composition and mechanism of the novel Chinese formula Nao Tan Qing [16]. Therefore, we applied network pharmacology to a comprehensive analysis of CRD ingredients and their possible action targets as well as targets related to AD. The relevant pathways were determined through KEGG and GO analysis. Molecular docking is often used to quickly and efficiently predict the binding ability of drug molecules to target proteins and the binding ability of dihydroquercetin to acetylcholinesterase and butyrylcholinesterase was analyzed to predict its therapeutic potential for Alzheimer's disease [17]. The binding ability of CRD ingredients to AD-related targets was verified through molecular docking. The model animal used in this study was 3 × Tg mice, which are a common model animal in studies on AD [18, 19].

**Fig. 1:**
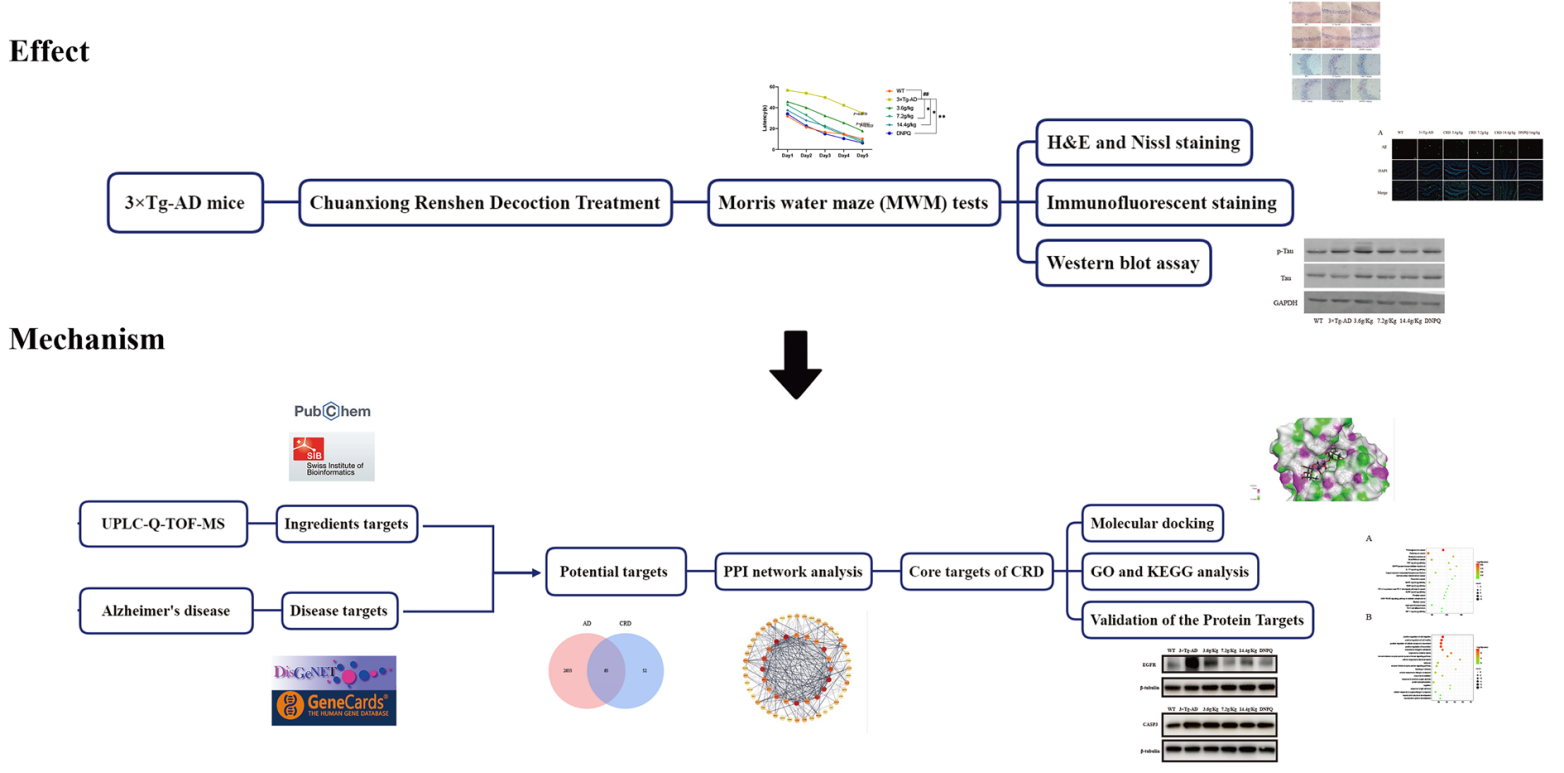
A comprehensive strategy diagram of the behavioral testing, chemical ingredient analysis, target prediction and network calculation for investigating the mechanism of action of CRD on AD

In summary, we aimed to explore the effect of CRD in 3 × Tg-AD mice, clarify the possible active ingredients against AD and analyze the possible mechanism of CRD to provide a basis for further development. The flow chart of this study is shown in Fig. 1.

## Materials and methods

### Preparation of CRD

CRD was prepared from a total of 4320 g of *Conioselinum anthriscoides 'Chuanxiong' (Chuanxiong), Panax ginseng C.A. (Renshen), Pueraria montana var. lobata (Willd.) Maesen & S.M. Almeida ex Sanjappa & Predeep (Gegen), Ginkgo Biloba L. (Yinxingye),* and *Reynoutria multiflora (Thunb.) Moldenke (Heshouwu)* in a 1:2:2:2:2 ratio*.* All herbs were obtained from Huadong Medicine Co., Ltd. (Zhejiang, China, Batch No. 20210208) and were fully validated by Associate Professor Ying Yao (Department of Pharmacognosy, School of Pharmacy, Hangzhou Medical College) according to the Chinese Pharmacopoeia 2015. The herbs were slightly crushed and placed in a flask with 8 volumes (W/V) of 60% ethanol and subjected to reflux heating for 2 h, followed by filtering. The alcohol extract was filtered, placed in a refrigerator at 4 °C for 24 h, and filtered again to obtain the drug solution. This solution was concentrated to 1.44 g/mL (14.4 g/kg) by a rotary evaporator and stored at the Zhejiang Institute of Traditional Chinese Medicine. When used in animal experiments, the solution was diluted to 0.72 g/mL and 0.36 g/mL with distilled water, which were equivalent to CRD 7.2 g/kg and 3.6 g/kg, respectively.

### Quality control markers of CRD

With reference to the requirements of the Chinese Pharmacopoeia 2020 regarding the above Chinese medicinal materials, we selected ferulic acid, ginsenoside Rg1, puerarin, ginkgolide A and emodin as the quality control markers for *Conioselinum anthriscoides 'Chuanxiong' (Chuanxiong), Panax ginseng C.A. (Renshen), Pueraria montana var. lobata (Willd.) Maesen & S.M. Almeida ex Sanjappa & Predeep (Gegen), Ginkgo Biloba L. (Yinxingye),* and *Reynoutria multiflora (Thunb.) Moldenke (Heshouwu)*, respectively.

### Animals and drug administration

Experimental 3 × Tg-AD transgenic mice (*APPSwe, tauP301L, PSEN1dE9*, females) and C57BL/6 background mice (25 ± 5 g) were placed separately in plastic cages at 22 ± 1 °C and 55% ± 5% humidity according to the 12-h light–dark cycle. All procedures involving experimental animals were performed according to the guidance on the treatment of the experimental animals issued by the Ministry of Science and Technology of the People's Republic of China and were also approved by the animal experiment ethics committee of Zhejiang University of Traditional Chinese Medicine (Ethics Approval No. ZSLL-2018-045). Five-month-old 3 × Tg-AD transgenic mice were randomly divided into 5 groups with 9 mice in each group: the 3 × Tg-AD group, three CRD groups (3.6 g/kg/d; 7.2 g/kg/day; 14.4 g/kg/d), and the donepezil treatment group (1 mg/kg/d, DNPQ, batch 1705080, Eisai Pharmaceutical Co., Ltd., Benxi, Liaoning, China). Nine C57BL/6 mice were used as the wild-type (WT) group. We used the WT group as the normal control group, the 3 × Tg-AD group as the model control group, and the donepezil group as the positive control group. The CRD groups were intragastrically administered (0.1 mL/10 g) once a day for 4 months. The WT group and 3 × Tg-AD group were given the same amount of normal saline by gavage.

### Morris water maze (MWM) tests

At 1, 2, 3 and 4 months after administration, all mice were subjected to the MWM test for 5 days to evaluate their learning and memory ability. The water temperature was kept at 22 ± 0.5 °C during the test.

#### Place navigation test

The learning and access memory abilities of mice were evaluated by a place navigation test for 5 days. The day before the experiment, the mice were put into the water to swim freely for 60 s to familiarize themselves with the experimental environment. The tank was divided into four quadrants, and four fixed points were chosen as starting points. A mouse was placed in the water at one of the four points. The escape latency and the time to reach the platform were recorded.

#### Spatial probe test

On the sixth day of the experiment, a spatial probe test was performed. The hidden platform was removed, and a mouse was placed in a tank with its face toward the wall in a randomly selected quadrant. The swimming trajectory and the number of times the mice crossed the original platform were recorded within 60 s.

### Brain tissue staining

H&E staining was performed as follows: 30 min after the last administration, the mice were anesthetized with 3% pentobarbital sodium intraperitoneally. Cardiac perfusion was performed before brain tissue staining. The left side of the brain was separated and fixed in 10% neutral formaldehyde fixative for 24 h. After the brain tissue was dehydrated and cleared, it was embedded in paraffin and sectioned. The tissue was dried at 65 ℃, dewaxed with xylene twice for 5 min, and washed with distilled water. Slides were placed in hematoxylin staining solution for 5 min after washing for 5 min. Differentiation was performed using acid alcohol for 30 s, and tap water immersion was performed for 15 min, followed by eosin staining for 2 min. Finally, the dehydration, clearing, rinsing and mounting were performed as follows: 95% ethanol for 1 min, ethanol for 3 min, anhydrous ethanol for 5 min (repeat twice), xylene for 5 min (repeat twice), and neutral balm for mounting [20].

Nissl staining was performed as follows: (1) dewaxing paraffin sections; (2) distilled water washing; (3) soaking in 1% toluidine blue aqueous solution in a constant temperature box at 50–60 °C and for 30 min; (4) distilled water washing; (5) rapid differentiation in 95% ethanol; and (6) anhydrous ethanol dehydration, xylene treatment for transparency, and neutral gum sealing [20].

### Immunofluorescent staining

Brain sections were roasted at 60 °C for 2 h and dewaxed. After washing, the sections were repaired with citrate sodium buffer in a microwave oven. Antibodies against Aβ (amyloid 1–16, 1:100, 803001, BioLegend) were added and incubated at 4 °C overnight following BSA blocking. Then, the slices were incubated with goat anti-mouse secondary antibody at room temperature for 1 h. Five different fields were randomly selected for photos under a 10× objective lens. All images were statistically analyzed by ImageJ software, and the degree of immunofluorescence staining was reflected by the cumulative optical density of Aβ plaques. Cumulative optical density (IOD) refers to the sum of fluorescence intensity in an image. The formula is IOD (cumulative optical density) = ∑area (positive expression area) × density (average fluorescence intensity).

### Western blot assay

Lysates of the hippocampus in brain tissues were collected, and the protein concentrations were determined by the BCA protein assay (Beyotime, China). Equal amounts of protein were loaded in each lane and separately subjected to SDS‒PAGE before transfer onto PVDF membranes (Millipore, Massachusetts, USA). After blocking with 5% skim milk for 1.5 h at 37 °C, the membrane was incubated with the indicated primary antibodies at 4 °C overnight and subsequently with the respective near-infrared dye-tagged secondary antibodies for 1 h at 37 °C. Antibodies were purchased from Thermo Fisher (anti-tau (phospho-Ser202 Thr205), MN1020), Immunoway (anti-tau, YT4546), and Hangzhou Huaan Biotechnology Co., LTD (GAPDH EM1101). Image acquisition and documentation of the blots were performed by an Odyssey double color infrared laser imaging system (LI-COR, Nebraska, USA). The analysis software of the Odyssey two-color infrared laser imaging system was used for analysis. The equation was as follows: relative gray value = (sample gray value/sample internal reference)/(control gray value/control internal reference). After obtaining the core target through network pharmacological prediction, we verified it through western blotting. The antibodies used were anti-*EGFR* (ab52894, Abcam), anti-*CASP3* (14220S, Cell Signaling Technology) and anti-β-tubulin (AM1031A, Abcepta). An Amersham ImageQuant 800 system (Cytiva, China) and ImageJ software were used to quantify the expression of EGFR and CASP3.

### Preparation of homogenates from brain tissue and serum

Ten 3 × Tg-AD mice (female mice in an SPF grade, weight 25 ± 2 g, 5 months old) were randomly assigned to the control group and CRD group (1.44 g/kg/d). Experimental mice were placed separately in plastic cages at 22 ± 1 °C and 55% ± 5% humidity according to the 12-h light–dark cycle (Ethics Approval No. ZJCLA-IACUC-20020056). The CRD group was intragastrically administered CRD once a day for 5 days with a gavage volume of 0.1 mL/10 g. The control group was given the same volume of distilled water in the same way. After the last administration, anesthesia was administered, the brain was removed by craniectomy, the brain tissue was repeatedly rinsed with normal saline until it became colorless, and the water droplets on the surface of the brain tissue were dried with paper. The processed brain tissue was accurately weighed and transferred to EP tubes, 3 volumes of normal saline was added, and homogenate beads were added for homogenization. Thirty minutes after the last drug administration, blood was taken from the abdominal aorta and allowed to stand at room temperature for 30 min, followed by centrifugation at 3000 rpm at 4 °C for 15 min. The supernatant was collected and stored in an EP tube at − 80 °C.

### UPLC-Q–TOF–MS analysis

#### Equipment and materials

The instruments and reagents used for mass spectrometry were as follows: SCIEX X-500R Quadrupole Time of Flight Mass Spectrometer (AB SCIEX, USA); TurboIonSpray ion source (AB SCIEX); Waters ACQUITY I-Class Plus UPLC Ultra-High-Performance Liquid Chromatography System (Waters); Thermo ST40R Low-temperature high-speed centrifuge (Thermo Fisher); IKA Miniature scroll mixing instrument (IKA Germany); AUW220D electronic balance (Shimadzu Company, Japan). Methanol, acetonitrile and formic acid (Merck, Germany), Milli-Q ultra-pure water (Millipore, USA); and other reagents were obtained in analytically pure form.

#### Preparation of CRD test solution

The preparation method of the original drug solution was performed as previously described. Then, 200 μL of the original drug solution (1.44 g/mL) was diluted in 800 μL of water and vortexed for 1 min to obtain a 0.384 g/mL solution. Then, 0.5 mL of 0.384 g/mL solution was mixed with 0.5 mL of methanol, vortexed for 1 min, and centrifuged at 14,000 rpm for 20 min. A 2 μL sample of the supernatant was obtained.

#### Preparation of standard curve solution

First, 200 μL of 1 mg/mL ginsenoside Rg1 standard solution, 200 μL of 1 mg/mL ferulic acid standard solution, 100 μL of 1 mg/mL emodin standard solution, 60 μL of 1 mg/mL ginkgolide A standard solution, and 60 μL of 1 mg/mL puerarin standard solution were placed in a centrifuge tube, and 40 μL of methanol was added. The mixture was vortexed to obtain the solution with concentration 1. The solution with concentration 1 was diluted by a factor of two. Then, 500 μL of the solution with concentration 1 was placed in a new centrifuge tube and diluted with 500 μL of methanol, and the same procedure was repeated to obtain standard solutions at seven concentrations. After centrifugation at 12,000 rpm/min for 20 min, the supernatant was collected and injected into the sample.

#### Preparation of homogenate of brain tissue and serum test solution

Brain tissue homogenate (200 μL) was mixed with methanol (800 μL), vortex shocked for 3 min, and centrifuged at 8000 rpm for 10 min. Next, 600 μL of supernatant was blown dry with nitrogen, dissolved in 200 μL of methanol, vortex shocked for 3 min, and centrifuged at 13,000 rpm for 10 min. A sample of the supernatant was taken for determination.

The serum was thawed at room temperature, 1.0 mL was placed in a centrifuge tube, and 20 μL of phosphoric acid was added accurately. Ultrasonic treatment was carried out for 1 min, vortex mixing was carried out for 30 s, and the samples were transferred to the SPE column, which previously activated and equilibrated with 3 mL of methanol and 3 mL of water in advance. The samples were washed with 3 mL of water, discarded, and eluted with 3 mL of methanol, and the eluent was collected and lyophilized. The residue was redissolved in 150 μL of methanol and centrifuged at 14,000 rpm at 4 ℃ for 15 min. The supernatant was used as the serum test solution.

#### UPLC-Q–TOF–MS conditions

The chromatographic conditions were as follows: an ACQUITY UPLC BEH C18 (150 × 2.1 mm, 1.7 μm) column with mobile phases of 0.1% formic acid acetonitrile (A) − 0.1% formic acid water (B); gradient elution procedure: 0–15 min, 95–50% B; 15–16.5 min, 50–30% B; 16.5–20 min, 30–20% B; 20–22.5 min, 20–10%; 22.5–23 min, 10–1% B; flow rate: 0.3 mL/min; injection tray temperature: 8 ℃; column temperature: 40 ℃; injection volume: 4 μL.

The mass spectrometry conditions were as follows: ion source gas 1 (Gas1): 55, ion source gas 2 (Gas2): 55, curtain gas (CUR): 35, source temperature: 600 ℃, ion spray voltage floating (ISVF): 5500 V/− 4500 V; TOF MS scan m/z range: 50–1500 Da, production scan m/z range: 25–1000 Da. The data were also decluttered by information-dependent acquisition (IDA) in high-sensitivity mode. Collision energy: 35 ± 15 eV; IDA: exclude isotopes within 4 Da, candidate ions to monitor per cycle: 12.

#### Identification of the ingredients

SCIEX OS (v2.0.1) software was used to collect and process the data. We first used the TCM MS/MS Library (TCM MS/MS Library contains more than 1000 secondary data of TCM compounds) as a database to identify CRD test solutions. Then, we used the identified ingredients as a self-built database to perform SCIEX OS software analysis of brain tissue homogenate of the CRD group and control group.

### Target collection

The potential targets of the ingredients in CRD were searched in SwissTargetPrediction [21], a web server that accurately predicts bioactive molecular targets based on the combination of 2D and 3D similarity measures of known ligands. A probability greater than 0.1 was considered to indicate a possible regulatory target of CRD ingredients. The targets related to AD were selected from the databases Disgenet and Genecards. Then, a Venn diagram was drawn to identify the intersection of ingredient-related targets and disease-related targets, which are potential targets for the treatment of CRD in AD.

### Protein‒protein interaction (PPI) network construction

Potential targets of CRD and AD were uploaded to STRING 11.0 (https://string-db.org/). The protein type was set to "Homo sapiens", and the minimum interaction score was 0.4. The results obtained from STRING were imported into Cytoscape V 3.8.2 software, and the core targets of the PPI network were determined by using the centriscape computing degree centrality (DC).

### Gene ontology and Kyoto encyclopedia of genes and genomes pathway enrichment analysis

Metascape was used to perform pathway enrichment and biological process annotation. Metascape perfectly makes up for the shortcomings of DAVID while retaining its advantages. The data are updated frequently to ensure timeliness and reliability. We entered the core potential targets of CRD ingredients into Metascape and selected "Homo sapiens" for enrichment analysis to examine the role of potential targets in gene function and signaling pathways.

### Molecular docking

In this study, we aimed to identify the interactions between ingredients and their targets and explore their binding patterns. Therefore, we selected the ingredients that could be absorbed and 20 core targets for molecular docking verification. The core ingredient PDB format was obtained from the UniProt database, and the X-ray crystal structures were obtained from the RCSB database. The LibDock module utilizes the molecular docking function of Discovery Studio 2016 (DS) to perform ingredient–target molecular docking. Then, we drew a heatmap of the core ingredients for molecular docking using the OMIC Studio website [22].

### Statistical analysis

All data are presented as the mean ± SD and were analyzed using SPSS 24.0 software (IBM Corp., Armonk, NY, USA). The data of each group were tested for normality: if the data were normally distributed, one-way ANOVA was used; if the equations were homogeneous, the LSD test was used; if the equations were not homogeneous, the Games-Howell (A) test was used; otherwise, the Kruskal‒Wallis H test method was used for analysis. Differences at *p* < 0.05 and *p* < 0.01 were considered to be significant.

## Results

### Results of CRD quality control

UPLC-Q–TOF–MS was used to analyze CRD and corresponding standards for quality control markers. After identification, all the markers were found. The content of the quality control markers in CRD met the requirements of the Chinese Pharmacopoeia 2020, as shown in Additional file 1: Table S1. Representative total ion chromatograms are shown in Additional file 2: Fig. S1.

### CRD significantly ameliorates learning and memory impairments in 3 × Tg-AD mice

**Fig. 2:**
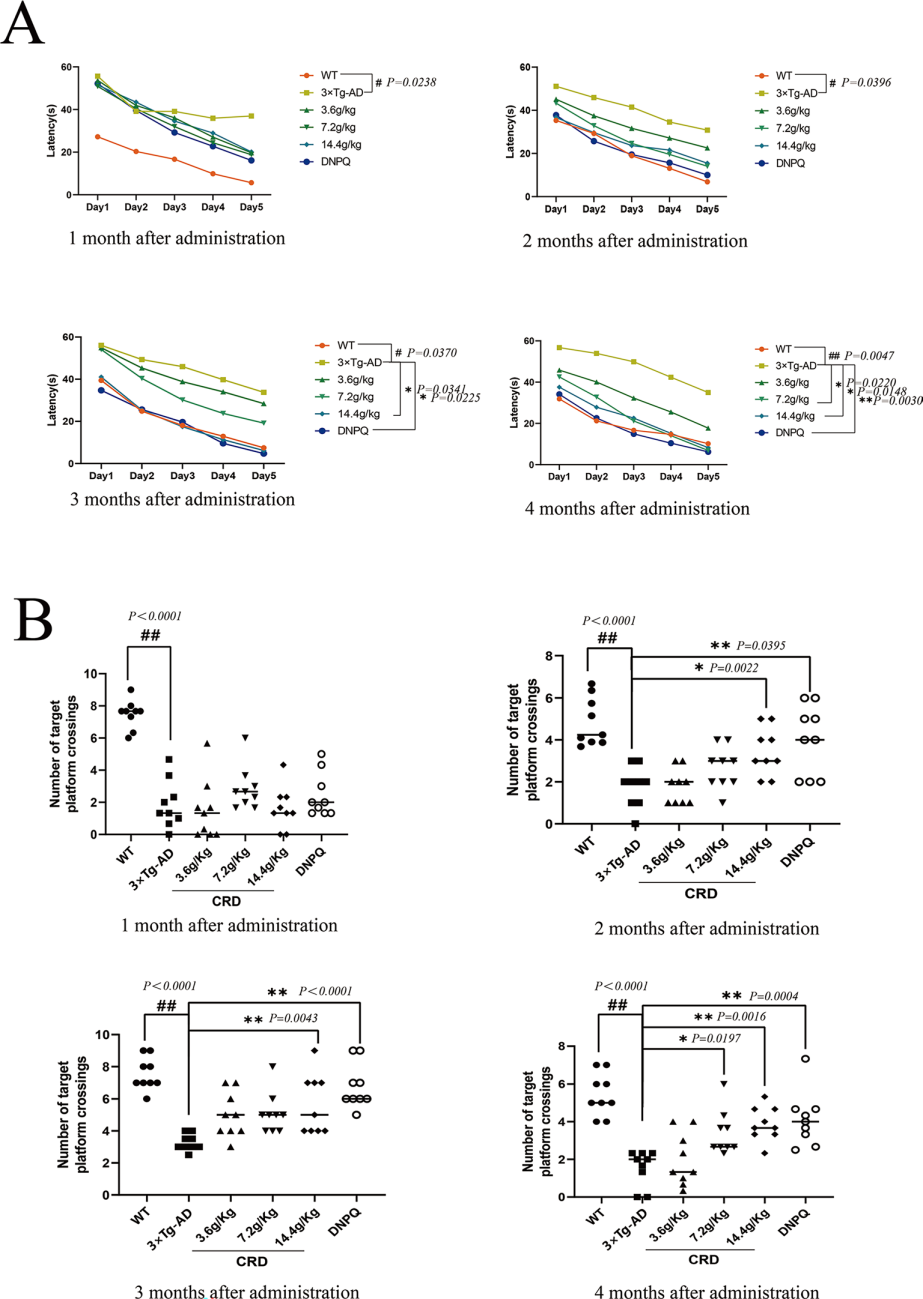
CRD ameliorates learning and memory impairments in 3 × Tg-AD mice. **A** The time needed to reach the hidden platform. **B** The number of crossings over the area where the escape platform was previously located. Data are expressed as the mean from 9 mice per group. ^*#*^*p* < 0.05, ^*##*^*p* < 0.01 compared with the WT group. *p < 0.05, **p < 0.01 versus the 3 × Tg-AD group

We examined the effects of CRD on learning and memory ability in 3 × Tg-AD mice using the MWM test. As shown in Fig. 2, the escape latency of 3 × Tg-AD mice (9 months old) was significantly longer than that of WT mice. The number of platform crossings was significantly decreased in the 3 × Tg-AD group (*p* < 0.01). After 4 months of CRD treatment, the escape latency of CRD groups was significantly shortened in the 3.6 g/kg (*p* < 0.01), 7.2 g/kg (*p* < 0.01) and 14.4 g/kg groups (*p* < 0.01), and the number of platform crossings was significantly increased in the 7.2 g/kg and 14.4 g/kg groups (*p* < 0.01). Together, these results suggested that CRD can ameliorate spatial learning and memory impairment in 3 × Tg-AD mice.

### CRD attenuates neuropathological changes in 3 × Tg-AD mice

**Fig. 3:**
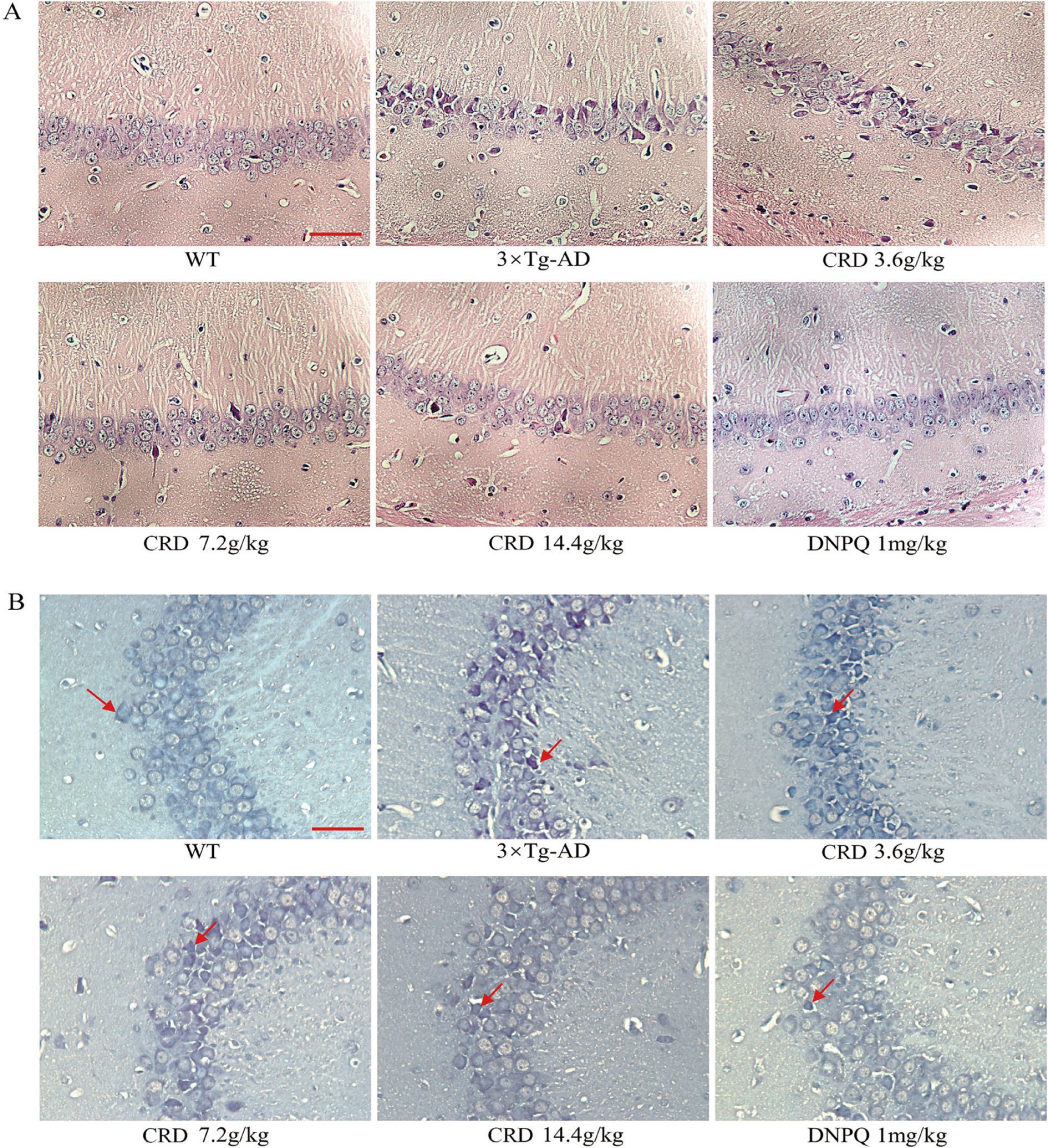
CRD attenuates neuropathological changes in 3 × Tg-AD mice. Sections obtained from the hippocampus were stained with HE (**A**) and Nissl staining (**B**). Representative images of three mice from each group. Scale bar, 50 μm

H&E staining results showed that in the hippocampus of the WT group, the neurons had an ordered arrangement, clear nuclei, distinct nucleoli and rich cytoplasm, with light staining. In 3 × Tg-AD mice (9 months old), neurons were loosely arranged, and their contents were concentrated with deep staining. The structure of the neurons was not clear, and the nucleus was pyknotic. After 4 months of CRD treatment, mice in the CRD groups had an ordered arrangement of neurons that were lightly stained and rich in cytoplasm relative to the 3 × Tg-AD group (Fig. 3A).

Nissl staining results showed that the CA1 region neurons in the hippocampus of the WT group were abundant, with clear kernels and lightly stained nuclei. In contrast, the neuronal tissue observed in the 3 × Tg-AD group (9 months old) was disorganized, swollen and deformed, with condensates and deeply stained nuclei. After 4 months of CRD treatment, Nissl corpuscles increased, and nuclear hyperchromatism decreased (Fig. 3B).

### CRD significantly reduced Aβ expression in the brain of 3 × Tg-AD mice, but tau phosphorylation levels did not change

**Fig. 4:**
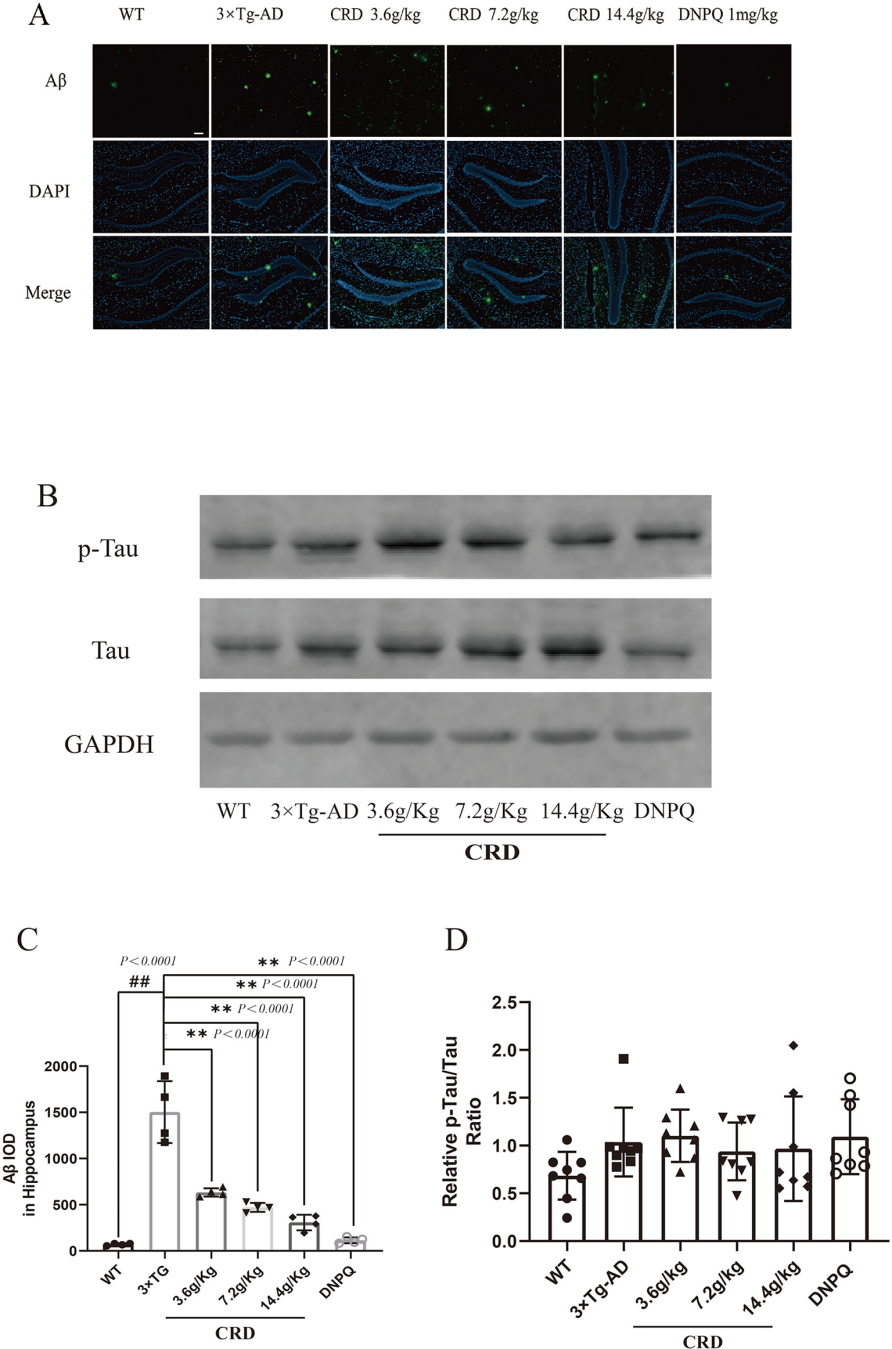
CRD reduces Aβ plaque formation in the hippocampus, but the Tau phosphorylation levels in the brain do not change. Immunofluorescent staining of Aβ plaques in the hippocampus (**A**). Scale bar, 100 μm. Cumulative optical density (IOD) of Aβ plaques in the hippocampus (**C**), each group n = 4. Representative blots of the hippocampus of 3 × Tg-AD mice administered CRD or saline solution were analyzed by immunoblotting (**B**). Quantification of data from the upper panel (n = 4 mice) (**D**). Data are expressed as the mean ± SD from 4 mice per group. ^*#*^*p* < 0.05, ^*##*^*p* < 0.01 compared with the WT group. *p < 0.05, **p < 0.01 versus the 3 × Tg-AD group

There is increasing evidence that Aβ aggregation in the brain is a pathogenic factor in AD. The immunofluorescent staining results showed that the cumulative optical density (IOD) of Aβ was weak in the hippocampus (Fig. 4A) of WT mice, whereas the Aβ cumulative optical density (IOD) was significantly increased in 3 × Tg-AD mice compared with WT mice. After 4 months of treatment, the Aβ cumulative optical density (IOD) was significantly reduced in the CRD groups (*p* < 0.01) in the hippocampus (Fig. 4C). We then examined whether CRD can modulate tau phosphorylation, another hallmark of AD. We detected the ratio between the gray value of the p-tau protein band and the gray value of tau protein by western blot. As shown in Fig. 4D, we found no statistically significant difference in tau phosphorylation levels between the CRD groups and the 3 × Tg-AD group. Moreover, there was no significant difference in tau phosphorylation between the WT group and the 3 × Tg-AD group. We concluded that tau phosphorylation did not occur in 9-month-old 3 × Tg-AD mice. The improvement in the phosphorylation level of tau protein by CRD in 3 × Tg-AD mice needs further study in older mice.

### Separation and identification of ingredients of CRD

A total of 95 chemical ingredients were identified in CRD by using the UPLC-Q–TOF MS system. The TICs are shown in Additional file 3: Fig. S2, Additional file 4: Fig. S2 and Additional file 5: Fig. S2. The ingredients included puerarin, ligustilide and ginsenoside Rg1 (Table 1). After analyzing and comparing the ingredients in the serum of the CRD group and the control group, 25 differential ingredients were obtained. These included levistilide A and ginsenoside-ro (Table 2). There were 20 ingredients in the brain tissue homogenate of the CRD group, including ferulic acid and panaxydol (Additional file 6: Table S2), and 18 ingredients in the brain tissue homogenate of the control group (Additional file 7: Table S3). After analyzing and comparing the ingredients of brain tissue homogenate of CRD group and control group, 5 differential ingredients in the homogenate of brain tissue were obtained. These included quinic acid, rutin, ferulic acid, ginsenoside Rg1, and panaxydol (Table 3). The five ingredients in brain tissue were predicted by the SwissTargetPrediction database. A total of 126 targets were obtained, and 117 targets were obtained after removing the repeated targets.

**Table 1:** Information about ingredients in CRD by using UPLC-Q–TOF–MS

No	Retention time	Ingredient name	Mode	Formula	Precursor mass	Found at mass	Mass error (ppm)	MS/MS fragment ions (m/z)
1	1.08	Histidine	NEG	C_6_H_9_N_3_O_2_	154.062	154.0619	− 2.1	90.0460, 80.0380, 154.0062
2	1.09	l(+)-Arginine	NEG	C_6_H_14_N_4_O_2_	173.104	173.1042	− 1.3	131.0824, 41.0143
3	1.11	Aspartic acid	NEG	C_4_H_7_NO_4_	132.03	132.03	− 1.5	88.0400, 41.9993, 71.0135
4	1.16	Betaine	POS	C_5_H_11_NO_2_	118.086	118.0861	− 1.2	58.0648, 118.0863
5	1.2	Trigonelline	POS	C_7_H_7_NO_2_	138.055	138.0548	− 1.4	92.0485, 138.0548, 78.0337
6	1.24	Quinic acid	NEG	C_7_H_12_O_6_	431.098	431.0988	− 1	191.0560, 85.0296, 93.0347
7	1.24	Proline	POS	C_5_H_9_NO_2_	116.071	116.0705	− 1.1	70.0651, 116.0705
8	1.5	Shikimic acid	NEG	C_7_H_10_O_5_	173.046	173.0454	− 0.6	93.0347, 85.0299, 111.0091
9	1.57	Adenine	POS	C_5_H_5_N_5_	136.062	136.0615	− 2	119.0353, 136.0617, 92.0242
10	1.64	Cytidine	NEG	C_9_H_13_N_3_O_5_	242.078	242.078	− 1	109.0406, 81.0450, 41.9983
11	1.74	Nicotinic acid	POS	C_6_H_5_NO_2_	124.039	124.0392	− 1	124.0391, 78.0337, 80.0409
12	1.92	Nicotinamide	POS	C_6_H_6_N_2_O	123.055	123.0551	− 1.9	80.0493, 123.0548, 53.0385
13	1.93	Citric acid	NEG	C_6_H_8_O_7_	191.02	191.0196	− 0.6	111.0089, 87.0087, 85.0296
14	2.1	Vitamin B6	POS	C_8_H_11_NO_3_	170.081	170.0811	− 0.5	134.0602, 152.0707, 94.0649
15	2.38	Amber acid	NEG	C_4_H_6_O_4_	117.019	117.0194	0.2	73.0299, 99.9261, 117.0200
16	2.54	Adenosine	NEG	C_10_H_13_N_5_O_4_	266.089	266.0896	0.5	134.0467, 107.0238
17	2.64	Guanosine	NEG	C_10_H_13_N_5_O_5_	282.084	282.0845	0.4	150.0425, 133.0157, 108.0204
18	2.78	Gallic acid	NEG	C_7_H_6_O_5_	169.014	169.0142	− 0.1	125.0246, 79.0188, 169.0155
19	3.01	Gastrodin	NEG	C_13_H_18_O_7_	285.098	285.098	0.2	123.0447, 105.0368, 77.0393
20	3.38	Phenprobamate	NEG	C_9_H_11_NO_2_	164.072	164.0717	0.2	103.0553, 72.0094
21	3.38	Phenylalanine	POS	C_9_H_11_NO_2_	166.086	166.0861	− 0.9	120.0804, 103.0543, 77.0386
22	4.02	Protocatechuic acid	NEG	C_7_H_6_O_4_	153.019	153.0194	0.4	109.0298, 153.0186
23	4.15	Hydroxytyrosol	NEG	C_8_H_10_O_3_	153.056	153.0558	0.6	123.0453, 153.0512
24	4.5	l-Tryptophan	NEG	C_11_H_12_N_2_O_2_	203.083	203.0825	− 0.7	116.0508, 203.0818, 74.0251
25	4.53	Higenamine	NEG	C_16_H_17_NO_3_	270.114	270.1131	− 1.8	162.0557, 160.0442
26	5.03	Protocatechuic aldehyde	NEG	C_7_H_6_O_3_	137.024	137.0244	− 0.2	136.0160, 108.0218, 92.0280
27	5.08	Vitexin	NEG	C_21_H_20_O_10_	431.098	431.0988	0.9	311.0563, 431.0984, 283.0612
28	5.18	Vaccarin	POS	C_32_H_38_O_19_	727.208	727.2086	0.8	595.1675, 727.2072, 313.0713
29	5.19	Chlorogenic acid	NEG	C_16_H_18_O_9_	353.088	353.0876	− 0.7	191.0558, 127.0410
30	5.24	Catechin	NEG	C_15_H_14_O_6_	289.072	289.0717	− 0.2	109.0297, 123.0458, 289.0758
31	5.41	*p*-Coumaric acid	NEG	C_9_H_8_O_3_	163.04	163.0399	− 0.8	119.0509, 93.0378
32	5.62	Secoisolariciresinol diglucoside	POS	C_32_H_46_O_16_.NH_3_	704.312	704.313	0.8	327.1579.137.0582
33	5.7	Esculetin	NEG	C_9_H_6_O_4_	177.019	177.0193	− 0.1	89.0290, 41.0029, 188.0178
34	5.8	Puerarin	NEG	C_21_H_20_O_9_	415.103	415.1031	− 0.8	267.0683, 295.0620, 415.1036
35	6.07	Vitamin B2	POS	C_17_H_20_N_4_O_6_	377.146	377.1457	0.3	243.0872, 377.1438
36	6.41	Pinoresinol diglucoside	NEG	C_32_H_42_O_16_.HCOOH	593.151	593.1511	− 0.6	357.1349, 519.1865
37	6.63	Daidzin	NEG	C_21_H_20_O_9_.HCOOH	415.103	415.1031	0.1	295.0620, 415.1036, 267.0683
38	6.83	Eleutheroside E	NEG	C_34_H_46_O_18_.HCOOH	787.267	787.2661	− 0.7	417.1547, 529.2046
39	6.99	Mulberroside A	NEG	C_26_H_32_O_14_	567.172	567.1719	0	243.0662, 567.1756
40	7.1	Typhaneoside	NEG	C_34_H_42_O_20_	769.22	769.2194	− 0.3	769.2190, 314.0435
41	7.3	Rutin	NEG	C_27_H_30_O_16_	609.146	609.1458	− 0.4	609.1461, 300.0276, 271.0244
42	7.41	Syringaldehyde	POS	C_9_H_10_O_4_	183.065	183.0651	− 0.5	95.0491, 77.0382, 140.0468
43	7.44	*p*-Anisic acid	NEG	C_8_H_8_O_3_	151.04	151.0401	0.2	107.0498, 92.0273
44	7.46	Daphnetin	POS	C_9_H_6_O_4_	179.034	179.0339	− 0.2	133.0283, 89.0388, 77.0392
45	7.49	Liquiritigenin	POS	C_15_H_12_O_4_	257.081	257.0809	0.4	137.0234, 147.0438, 257.0798
46	7.59	Scopoletin	POS	C_10_H_8_O_4_	193.05	193.0495	− 0.3	133.0283, 178.0246, 193.0494
47	7.6	Hyperin	NEG	C_21_H_20_O_12_	463.088	463.0879	− 0.6	300.0275, 463.0875, 271.0251
48	7.61	Isoquercitrin	POS	C_21_H_20_O_12_	465.103	465.1031	0.7	303.0501
49	7.62	Quercetin	POS	C_15_H_10_O_7_	303.05	303.0502	0.8	303.0497, 137.0604
50	7.64	2, 3, 5, 4′-Tetrahydroxystilbene-2-*O*-b-d-glucoside	NEG	C_20_H_22_O_9_	405.119	405.1187	− 1	243.0679, 137.0245, 173.0607
51	7.66	Ferulic acid	NEG	C_10_H_10_O_4_	193.051	193.0506	− 0.4	134.0371, 178.0275, 193.0507
52	7.86	6-Hydroxy-7, 8-dimethoxycoumarin	POS	C_11_H_10_O_5_	223.06	223.0602	0.5	223.0610, 190.0259, 162.0307
53	7.86	Isofraxidin	POS	C_11_H_10_O_5_	223.06	223.0602	0.5	223.0610, 162.0307, 107.0493
54	7.94	Ginkgolide B	NEG	C_20_H_24_O_10_.HCOOH	423.13	423.1293	− 0.6	125.0241, 367.1396, 423.1260
55	7.96	Aempferol-3-*O*-rutinoside	NEG	C_27_H_30_O_15_	593.151	593.1511	− 0.2	285.0402, 593.1514
56	8	Bilobalide	NEG	C_15_H_18_O_8_	325.093	325.0928	− 0.1	163.1128, 193.1235
57	8.09	Ginkgolide C	NEG	C_20_H_24_O_11_	439.125	439.1244	− 0.3	125.0245, 383.1359, 113.0248
58	8.11	Isorhamnetin-3-*O*-neohespeidoside	POS	C_28_H_32_O_16_	625.176	625.1763	0.1	317.0646
59	8.12	Isorhamnetin	POS	C_16_H_12_O_7_	317.066	317.0657	0.5	317.0664, 302.0421, 153.0179
60	8.23	1,5-Dicaffeoylquinic acid	NEG	C_25_H_24_O_12_	515.119	515.1194	− 0.1	191.0559, 353.0877
61	8.27	Calycosin	NEG	C_16_H_12_O_5_	283.061	283.0611	− 0.5	268.0371, 283.0604, 211.0397
62	8.3	Astragalin	NEG	C_21_H_20_O_11_	447.093	447.0928	− 1	284.0328, 447.0932, 255.0300
63	8.31	Pinoresinol-glucoside	NEG	C_26_H_32_O_11_	519.187	519.1868	− 0.7	151.0400, 357.1343
64	8.61	3-Hydroxymorindone	POS	C_15_H_10_O_6_	287.055	287.0551	0.2	287.0552, 153.0179
65	8.61	Kaempferol	POS	C_15_H_10_O_6_	287.055	287.0551	0.2	287.0552, 213.0537
66	9.63	Ginsenoside Rg1	NEG	C_42_H_72_O_14_.HCOOH	845.49	845.4899	− 0.6	799.4828, 637.4311, 161.0450
67	9.83	Ononin	NEG	C_22_H_22_O_9_.HCOOH	475.125	475.1242	− 0.8	267.0654, 252.0422
68	10.03	Daidzein	NEG	C_15_H_10_O_4_	253.051	253.0504	− 0.8	253.0506, 208.0533, 224.0478
69	10.04	Astragaloside I	NEG	C_45_H_72_O_16_.HCOOH	913.48	913.4794	− 0.9	913.4798, 914.4838
70	10.44	Ginkgolide A	POS	C_20_H_24_O_9_	409.149	409.1495	0.5	345.1328, 281.1172
71	10.88	Formononetin	NEG	C_16_H_12_O_4_	267.066	267.066	− 1	252.0429, 223.0405, 195.0449
72	11.22	Pectolinarigenin	NEG	C_17_H_14_O_6_	313.072	313.0715	− 0.8	283.0249, 227.0351, 298.0477
73	11.83	Naringenin	NEG	C_15_H_12_O_5_	271.061	271.0606	− 2.2	119.0501, 151.0034, 271.0592
74	12.17	Pseuoginsenoside F11	NEG	C_42_H_72_O_14_	799.485	799.4847	− 0.3	799.4858, 637.4295
75	12.39	Gypenoside XVII	POS	C_48_H_82_O_18_	947.557	947.5582	0.9	325.1132, 163.0602, 407.3671
76	12.66	Hydroxygenkwanin	NEG	C_16_H_12_O_6_	299.056	299.0558	− 0.9	255.0299, 284.0319, 299.0531
77	12.9	Ginsenoside Rg2	NEG	C_42_H_72_O_13_.HCOOH	829.495	829.495	− 0.6	783.4910, 101.0246
78	12.99	Ginsenoside-Ro	NEG	C_48_H_76_O_19_	955.491	955.4897	− 1.1	955.4885
79	12.99	Ursolic acid	POS	C_30_H_48_O_3_	457.368	457.3681	1	411.3602, 439.3555
80	13.02	Ginsenoside Rh1	NEG	C_36_H_62_O_9_.HCOOH	683.438	683.4371	− 0.7	683.4394, 637.4381, 475.3804
81	13.24	Amentoflavone	NEG	C_30_H_18_O_10_	537.083	537.0822	− 1	537.0828, 375.0496, 417.0616
82	14.28	Parthenolide	POS	C_15_H_20_O_3_	249.149	249.1485	− 0.1	119.0856, 145.1012
83	15.64	Saikosaponin C	NEG	C_48_H_78_O_17_	925.517	925.5155	− 1.2	925.5156
84	15.82	Neobavaisoflavone	NEG	C_20_H_18_O_4_	321.113	321.1128	− 1.3	321.1128, 265.0505, 305.0809
85	16.24	3-*N*-Butyl-4,5-dihydrophthalide	POS	C_12_H_16_O_2_	193.122	193.1218	− 2.5	91.0537, 77.0380, 137.0593
86	16.43	Raddeanin A	NEG	C_47_H_76_O_16_	895.506	895.5047	− 1.5	895.5055
87	16.86	Panaxydol	POS	C_17_H_24_O_2_	261.185	261.1847	− 0.6	77.0385, 105.0334
88	17.32	Emodin	NEG	C_15_H_10_O_5_	269.046	269.0456	0.2	269.0462, 225.0562, 241.0513
89	17.45	Ligustilide	POS	C_12_H_14_O_2_	191.107	191.106	− 3.3	91.0543, 79.0538, 191.1058
90	17.75	Costunolide	POS	C_15_H_20_O_2_	233.154	233.1534	− 0.9	177.0907, 91.0538
91	17.76	Pregnenolone	POS	C_21_H_32_O_2_	317.248	317.2473	− 0.7	159.1179, 281.2262, 317.2456
92	18.3	Gingerglycolipid B	NEG	C_33_H_58_O_14_.HCOOH	723.381	723.3809	0.1	397.1360, 677.3745, 279.2328
93	19.26	Reserpine	POS	C_33_H_40_N_2_O_9_	609.281	609.2811	0.7	609.2808, 397.2138, 195.0642
94	19.33	Patchouli alcohol	POS	C_15_H_24_	205.195	205.1949	− 0.9	93.0697, 121.1008
95	19.63	Levistilide A	POS	C_24_H_28_O_4_	381.206	381.206	− 0.2	191.1065, 149.0598

**Table 2:** Information about ingredients of CRD that identified in the serum by using UPLC-Q–TOF–MS

NO	Retention time	Ingredient name	Mode	Formula	Precursor mass	Found at mass	Mass error (ppm)	MS/MS fragment ions (m/z)
1	1.17	Betaine	POS	C_5_H_11_NO_2_	118.086	118.0862	− 0.4	72.0806, 55.0541
2	1.22	Quinic acid	NEG	C_7_H_12_O_6_	191.056	191.0559	− 1.2	111.0094, 191.0566, 85.0299
3	1.23	Proline	POS	C_5_H_9_NO_2_	116.071	116.0705	− 0.8	70.0652
4	3.39	Phenylalanine	POS	C_9_H_11_NO_2_	166.086	166.086	− 1.4	103.0545, 120.0808, 77.0388
5	4.05	Protocatechuic acid	NEG	C_7_H_6_O_4_	153.019	153.019	− 2	109.0308, 108.0211, 91.0178
6	4.54	l-Tryptophan	NEG	C_11_H_12_N_2_O_2_	203.083	203.0825	− 0.4	116.0505, 142.0665, 74.0250
7	5.07	Vitexin	NEG	C_21_H_20_O_10_	431.098	431.0981	− 0.2	311.0558, 286.0611, 431.1002
8	5.42	*p*-Coumaric acid	NEG	C_9_H_8_O_3_	163.04	163.0398	− 1.8	34.9693, 118.9953, 145.8898
9	5.79	Puerarin	NEG	C_21_H_20_O_9_	415.103	415.1036	− 0.3	267.0667, 295.0611, 415.1039
10	6.63	Daidzin	NEG	C_21_H_20_O_9_	461.109	461.1086	− 0.8	253.0503, 415.1025
11	7.25	Rutin	NEG	C_27_H_30_O_16_	609.146	609.1444	− 2.9	609.1455, 300.0271
12	7.45	Daphnetin	POS	C_9_H_6_O_4_	179.034	179.0333	− 3.3	136.0202, 179.0629
13	7.59	Hyperin	NEG	C_21_H_20_O_12_	463.088	463.0889	1.6	307.0765, 143.0458, 99.0551
14	7.64	2,3,5,4′-Tetrahydroxystilbene-2-*O*-b-d-glucoside	NEG	C_20_H_22_O_9_	407.134	407.1334	− 0.8	243.0643
15	7.65	Ferulic acid	POS	C_10_H_10_O_4_	195.065	195.0651	− 0.5	145.0284, 89.0385, 195.0649
16	7.88	Ginkgolide B	NEG	C_20_H_24_O_10_	469.135	469.1334	− 3.6	209.9080, 423.1232, 221.0594
17	8.56	3-Hydroxymorindone	POS	C_15_H_10_O_6_	287.055	287.055	0	241.0475, 287.0540
18	8.56	Kaempferol	POS	C_15_H_10_O_6_	287.055	287.055	0	241.0475, 287.0540, 213.0558
19	9.55	Ginsenoside Rg1	NEG	C_42_H_72_O_14_	845.49	845.4899	− 0.6	845.4947, 799.4892, 637.4372
20	10.02	Daidzein	NEG	C_15_H_10_O_4_	253.051	253.0508	− 0.9	253.0506, 223.0400, 208.0527
21	10.4	Ginkgolide A	POS	C_20_H_24_O_9_	409.149	409.1495	0.5	409.1489, 253.1234, 355.1101
22	12.91	Ginsenoside-Ro	NEG	C_48_H_76_O_19_	955.491	955.4904	− 0.5	955.4895, 793.4201
23	16.15	*N*-Butyl-4,5-dihydrophthalide	POS	C_12_H_16_O_2_	193.122	193.1222	− 0.6	93.0703, 77.0369, 147.1162
24	17.4	Ligustilide	POS	C_12_H_14_O_2_	191.107	191.1064	− 1.1	91.0547, 78.0388, 191.1087
25	19.54	Levistilide A	POS	C_24_H_28_O_4_	381.206	381.2067	1.7	191.1073, 381.2084

**Table 3:** Information about ingredients of CRD that identified in the homogenate of brain tissue by using UPLC-Q–TOF–MS

No	Ingredient name	Retention time	Mode	Formula	Precursor mass	Found at mass	Mass error (ppm)	Structural formula	CAS no.	MS/MS fragment ions (m/z)
1	Quinic acid	1.21	NEG	C_7_H_12_O_6_	191.056	191.0563	1.2		77-95-2	85.0298, 191.0564, 93.0349
2	Rutin	7.3	NEG	C_27_H_30_O_16_	609.146	609.1457	− 0.6		153-18-4	609.1482, 300.0263
3	Ferulic acid	7.69	POS	C_10_H_10_O_4_	195.065	195.0655	1.7		1135-24-6	163.0382, 77.0388, 45.0356
4	Ginsenoside Rg1	9.55	NEG	C_42_H_72_O_14_	845.49	845.49	− 0.5		22427-39-0	799.4855, 845.4914, 637.4328
5	Panaxydol	16.79	POS	C_17_H_24_O_2_	261.185	261.1846	− 1		72800-72-7	77.0384, 105.0329, 98.9866

### Potential targets of ingredients identified from brain tissue in treating AD

**Fig. 5:**
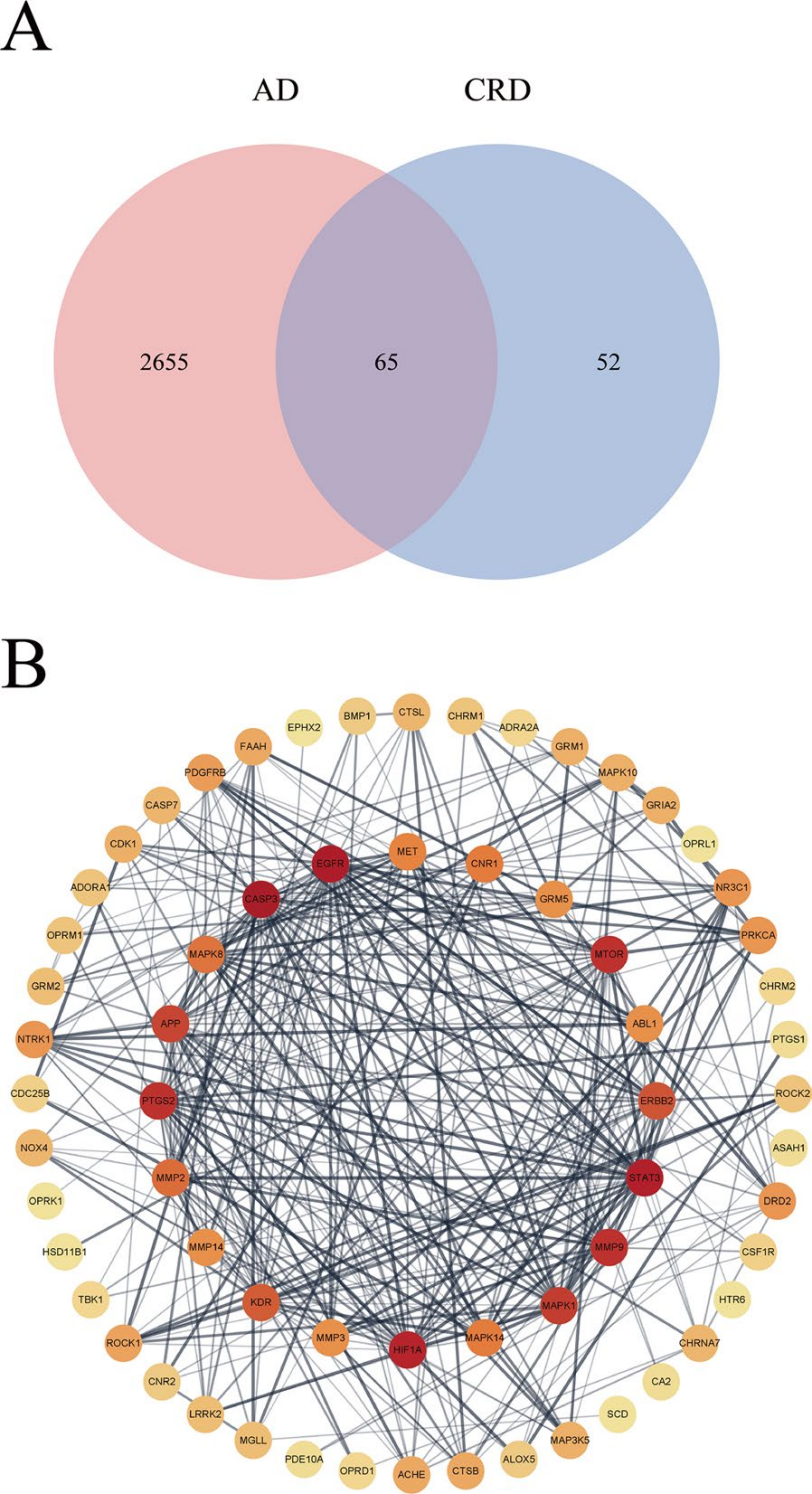
Network pharmacology of CRD. Venn diagram of related targets of CRD and Alzheimer’s disease (**A**). PPI network of 65 overlapping targets between drug and disease (**B**)

AD-related targets were collected from the human genome database. DisGeNET and GeneCard contained 3397 and 11,038 such targets, respectively. Genes repeated in both databases were selected as genes related to Alzheimer's disease, for a total of 2720 genes. Targets related to AD were intersected with targets related to ingredients, and 65 potential targets of CRD were obtained, as shown in Fig. 5A.

### Protein‒protein interaction network analysis of 65 potential targets

We uploaded 65 overlapping drug-disease targets in STRING, resulting in a PPI network consisting of 64 nodes and 354 edges. PPI network diagrams were drawn by Cytoscape (v3.8.2) software (Fig. 5B); the redder the color is, the higher the DC value. Except for *NQO2*, the remaining targets could also interact with other targets. The top 20 targets by degree value were considered core targets, and the highest degree targets were *CASP3* and *EGFR*.

### GO function and KEGG pathway enrichment analysis

**Fig. 6:**
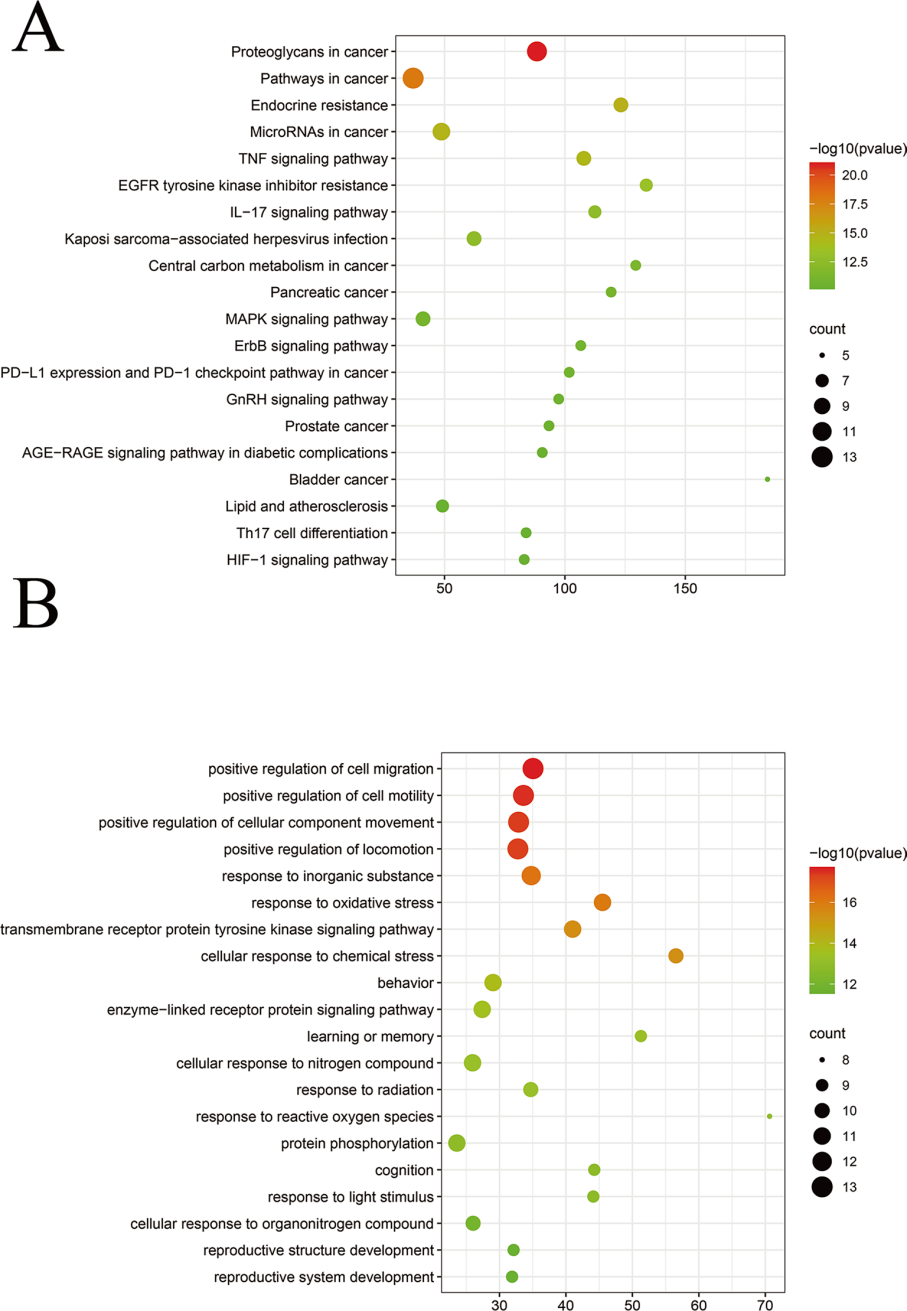
KEGG pathway and GO enrichment analyses of 20 target proteins. **A** Bubble chart of Gene Ontology (GO) functional enrichment of core targets. **B** Bubble chart of Kyoto Encyclopedia of Genes and Genomes (KEGG) enrichment of core targets

The enrichment results were selected under the conditions of *p* < 0.01, minimum count 3 and enrichment factor > 1.5. A total of 485 GO biological functions and 105 KEGG enrichment items were obtained. The KEGG pathways related to the treatment of AD included *EGFR* tyrosine kinase inhibitor resistance (hsa01521) and the MAPK signaling pathway (hsa04010) (Fig. 6B). The GO functions related to the treatment of AD included behavior (GO:0007610), response to inorganic substance (GO:0010035), and cognition (GO:0050890) (Fig. 6A).

### Molecular docking

**Fig. 7:**
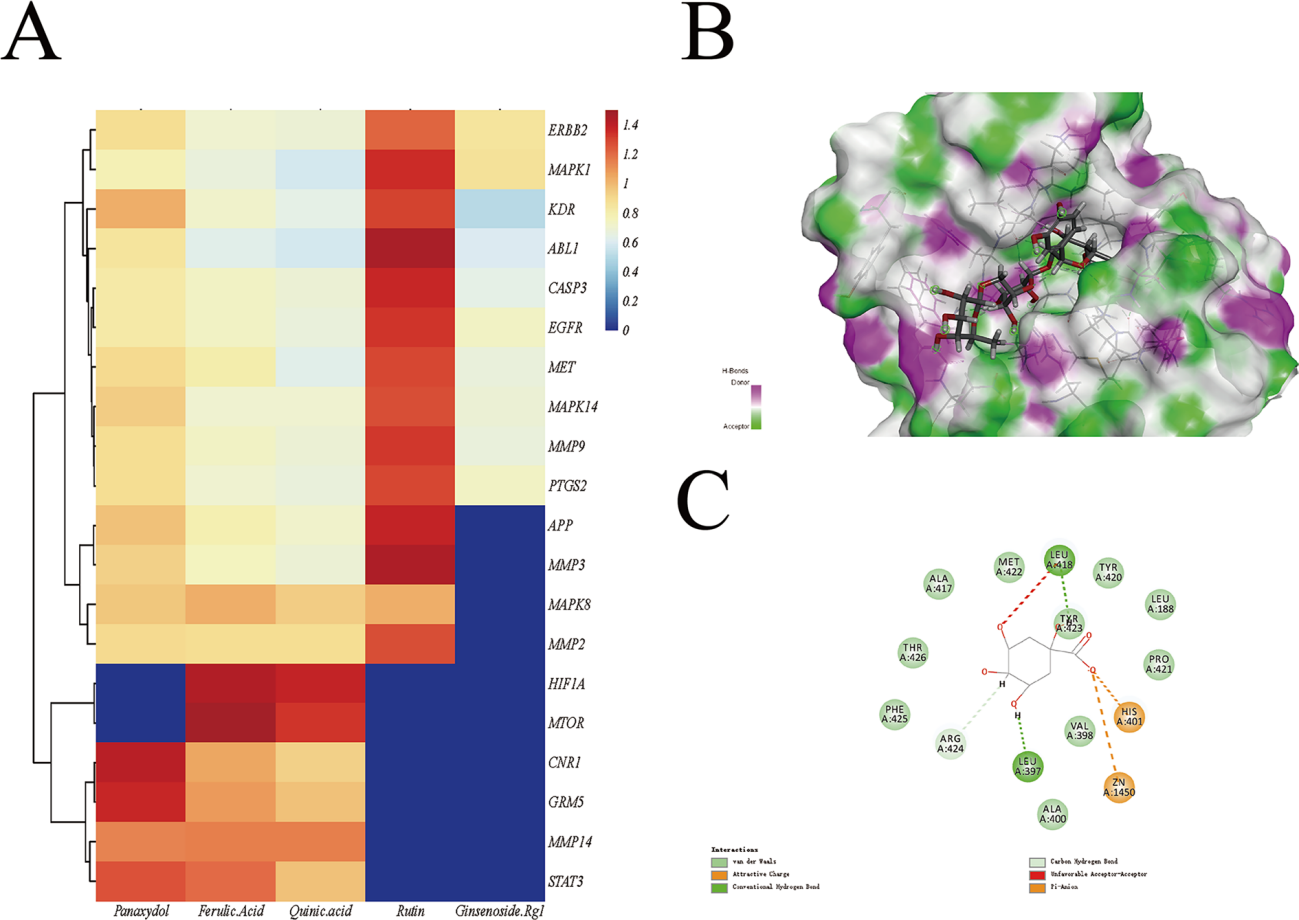
Results of molecular docking. **A** Ingredient-core target docking scores. **B** 3D and 2D graphs of rutin and MMP9 molecular docking results

TCM compounds have multiple ingredients and multiple targets. Therefore, we used DS (2016) software to dock core ingredients with core target molecules to explore their binding ability, and the higher the binding score between ligand and receptor was, the greater the possibility of interaction. The docking result of rutin with the protein *MMP9* (Fig. 7B, C) may be through hydrogen bonding, van der Waals forces and other forces. The scoring results of all docking are shown in Fig. 7A. The analysis of docking score results showed that the relationship between the above core ingredients and core indicators is consistent. *CASP3*, *EGFR* and *PTGS2* were the targets with high binding ability.

### CRD significantly decreased the expression of *EGFR* and *CASP3*

**Fig. 8:**
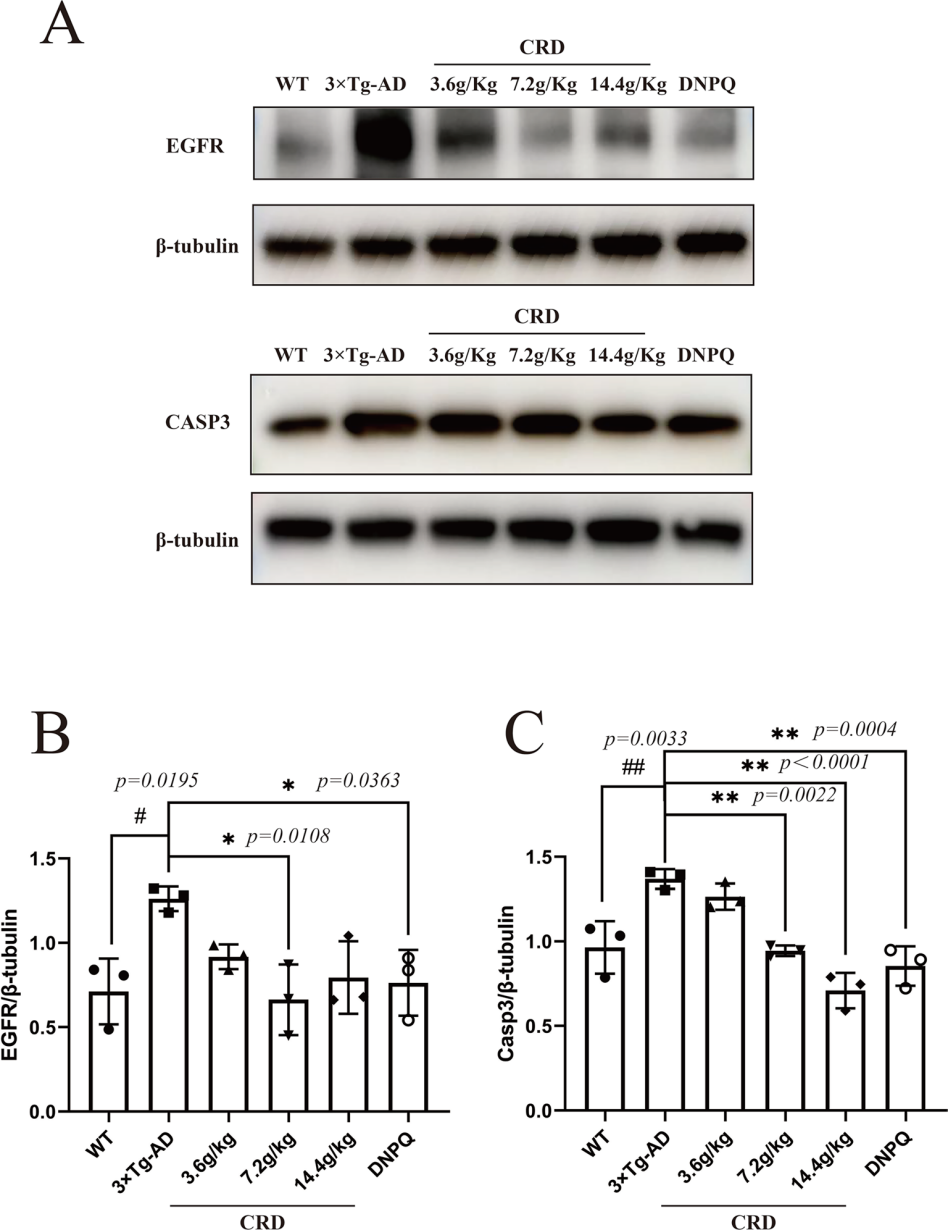
Validation of the protein targets. Representative images (**A**). The protein levels of *EGFR* and *CASP3* in hippocampal tissues from mice were measured by Western blotting (n = 3) (**B**, **C**)

According to PPI network analysis and molecular docking results, we selected the core targets *EGFR* and *CASP3* for western blot verification. *CASP3* and *EGFR* are essential proteins involved in the regulation of the p38 MAPK signaling pathway, which is widely accepted as a cascade contributing to neuroinflammation [23]. Representative western blotting images (Fig. 8A) and fold changes in the relative densitometric values of *CASP3* and *EGFR* are shown in Fig. 8B, C. Compared with the WT group, the expression of *EGFR* and *CASP3* in 3 × Tg-AD mice increased significantly (*p* < 0.05). The expression of *CASP3* was significantly decreased in the 7.2 g/kg CRD group (*p* < 0.01) and the 14.4 g/kg CRD group (*p* < 0.01), and the expression of *EGFR* was significantly decreased in the 7.2 g/kg CRD group (*p* < 0.01) compared with the model group. Western blotting results indicate that the therapeutic effect of CRD on AD may occur through downregulation of the expression of *CASP3* and *EGFR*.

## Discussion

The pathogenesis of AD is quite complicated; there is no unified conclusion in the academic community, and no efficient clinical treatment is available. Moreover, almost all drugs targeting a single target or single process, such as solanezumab, bapineuzumab and aducanumab, have not shown significant efficacy [24, 25]. In addition, the clinical trial of leuco-methylthioninium (LMTM) and methylthioninium (MT), Tau aggregation inhibitors designed to target Tau protein, showed no significant improvement in AD [26, 27]. Therefore, we believe Alzheimer's disease to be a complex disease involving multiple processes. Looking for anti-AD drugs regulated by multitarget networks has important practical significance and broad application prospects.

Here, the potential effects of CRD on learning and memory of spatial position and direction were assessed using the Morris water maze test. As anticipated, 3 × Tg-AD mice showed marked impairment in spatial learning and memory. However, CRD significantly ameliorated this deficit. In addition, the hippocampus, which is located under the cerebral cortex and is mainly responsible for short-term and long-term memory and spatial positioning, is the primary area damaged in the progression of AD [28]. Morphological examination showed that the arrangement of neurons in the DG area of the hippocampus was significantly improved after CRD treatment. Moreover, Nissl bodies are widely distributed in the cytoplasm of neurons and are mainly composed of parallel rough endoplasmic reticulum and polyribosomes, which are responsible for protein synthesis. When neurons are stimulated, Nissl bodies decrease. The Nissl bodies improved after CRD treatment, indicating that CRD can ameliorate neuronal injury in AD. In conclusion, we found that CRD not only ameliorated disease-related behaviors but also reduced Aβ plaques. However, there was no significant difference in the level of tau phosphorylation between the WT group and the 3 × Tg-AD group. We hypothesized that tau phosphorylation had not occurred in the brains of 9-month-old 3 × Tg-AD mice. Jackson Lab's description of triple rotation mice indicated that aggregates of conformationally altered and hyperphosphorylated tau were detected in the hippocampus of 12- to 15-month-old 3 × Tg-AD mice (https://www.jax.org/strain/004807). In summary, we conclude that CRD has a protective effect on hippocampal neuronal cells by reducing the expression of Aβ protein in the hippocampus and ultimately ameliorating the decreased learning and memory ability of the model mice.

We identified the ingredients in CRD through network pharmacology combined with UPLC-Q/TOF–MS analysis. By comparing the data on brain tissue homogenates from the drug CRD group and control group with the data in the self-built database, we identified the main active ingredients in CRD, including ferulic acid, quinic acid, rutin, ginsenoside Rg1 and panaxydol. Ferulic acid has been reported to significantly alter circulating levels of phenolic compounds, which are associated with improved cognitive function [29]. Studies have shown that quinic acid derivatives have potential as therapeutic agents in AD [30]. Some studies suggest that rutin may be a promising neuroprotective compound for the treatment of neurodegenerative diseases [31]. Ginsenoside Rg1 can also alleviate cognitive impairments and neuronal damage and reduce Aβ deposition. Panaxydol may play a beneficial role in normal brain aging and neurodegenerative diseases [32]. Among ingredients that enter the brain, ferulic acid and rutin or their derivatives have been shown to improve memory function [33, 34].

According to the theory of TCM, the various herbs in the compound CRD coordinate with each other to invigorate qi and promote blood circulation and can be used to treat AD patients with qi deficiency and blood stasis. This is consistent with our results using network pharmacology. For example, ferulic acid from *Conioselinum anthriscoides 'Chuanxiong' (Chuanxiong)*, panaxydol from *Panax ginseng C.A. (Renshen)* and *rutin from Ginkgo Biloba L. (Yinxingye)* enter the brain and jointly regulate the target CA2.

The ingredients we identified in the drug-containing serum included puerain, daizein, emodin and 2,3,5,4′-tetrahydroxystilbene-2-*O*-b-d-glucoside. Puerain and daizein are the main ingredients of *Pueraria montana var. lobata (Willd.) Maesen & S.M. Almeida ex Sanjappa & Predeep.* Some studies have suggested that puerarin has potential to relieve AD [11], and daidzein has a preventive effect on memory and learning dysfunction and on oxidative stress in ICV-STZ rats [35]. Emodin and 2,3,5,4′-tetrahydroxystilbene-2-*O*-b-d-glucoside are the main ingredients of *Reynoutria multiflora (Thunb.) Moldenke*, and some researchers have reported that 6.25 mg/kg emodin ameliorates cognitive impairment by 60–70% in AD mice [36]. A meta-analysis indicated that 2,3,5,4′-tetrahydroxystilbene-2-*O*-b-d-glucoside also plays a potential therapeutic role in AD [37]. How the ingredients of CRD that cannot enter the brain function in the treatment of AD needs further investigation.

The core targets for the treatment of AD identified by the PPI network analysis of CRD include *CASP3*, *EGFR*, *APP*, *CNR1*, *PTGS2* and *GRM5*. Studies have indicated that caspase-3 is a potential target for pharmacological therapy during early AD stages [38]. *EGFR* inhibition ameliorates Aβ toxicity [39]. APP is the amyloid-β (Aβ) peptide precursor protein that accumulates in the brains of individuals with AD-related pathology [40]. Overexpression of *CNR1* reduced neuronal injury in the rat hippocampus [41]. It has also been reported that selectively inhibiting phosphatidylinositol-4,5-bisphosphate hydrolysis, which is mediated by *GRM5*, rescues synaptic and spatial learning and memory deficits in APP/PS1 mice [42]. The results of molecular docking revealed that the proteins encoded by *ABL1*, *CASP3*, *EGFR*, *PTGS2* and *MAPK1* bound well with the possible active ingredients of CRD.

Some studies have shown that inhibition of *EGFR* in different AD animal models leads to antiamyloidogenic and autophagy enhancement effects [43–45], anti-neuroinflammatory, antioxidant, and anti-astrogliosis effects [44, 46]. Recent research shows that caspase-3 is the endogenous modulator of Aβ production, which is a novel, attractive and viable Aβ-lowering therapeutic target for AD [47]. Some studies have indicated the existence of a caspase-3-dependent mechanism that drives synaptic failure and contributes to cognitive dysfunction in AD [38]. Our results indicate that the therapeutic effect of CRD on AD may occur through downregulation of the expression of *CASP3* and *EGFR*.

**Fig. 9:**
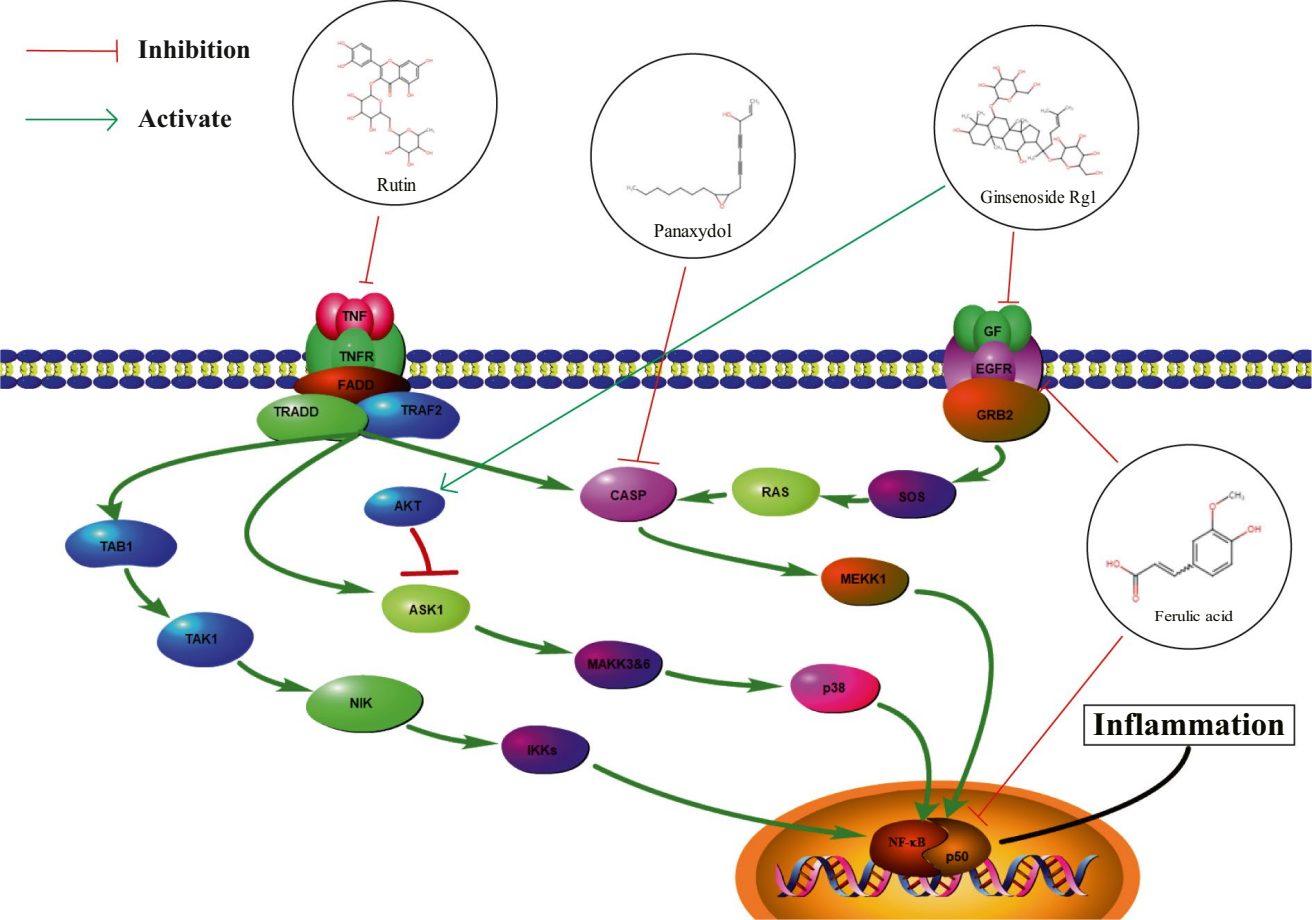
Potential mechanisms of CRD treatment of AD

Twenty core targets were enriched in GO and KEGG pathways, and 485 GO biological processes and 105 KEGG pathways were obtained. Some KEGG enrichment results were associated with Alzheimer's disease, such as IL-17 signaling pathway (hsa04657), TNF signaling pathway (hsa04668) and the MAPK signaling pathway (hsa04010). According to the GO enrichment analysis of core targets, 20 biological processes were identified. These processes include behavior (GO:0007610), response to oxidative stress (GO:0010035), and cognition (GO:0050890). Recent findings indicate that p38 MAPK signaling has been widely accepted as a cascade contributing to neuroinflammation, which is emerging as a new Alzheimer's disease treatment strategy [23]. Some studies have suggested that neuronal loss in AD is due to TNF-mediated necroptosis rather than apoptosis [48]. Results from in vitro experiments indicate that IL-17 might promote autophagy in neurons and thus induce Neurodegeneration [49, 50]. We found that among the five ingredients that can enter the brain, ferulic acid, rutin and panaxydol can inhibit the targets *EGFR*, *CASP3* and *TNF* in the MAPK pathway, respectively [51–53], while ginsenoside Rg1 can activate the targets *AKT* and *FGF2* [54, 55]. By combining the results of enrichment analysis with the ingredients of CRD that can enter the brain obtained by mass spectrometry, we obtained the possible mechanism of CRD in the treatment of AD (Fig. 9). We assume that the therapeutic effect of CRD on AD may be to reduce inflammation by inhibiting the MAPK pathway.

There are still some limitations in our study. First, the network pharmacology database is constantly updated, and it is possible that not all the regulatory targets of the ingredients are included in the current database. In addition, we verified that CRD significantly reduced *EGFR* and *CASP3* expression through western blotting, but which ingredients in CRD regulate *EGFR* and *CASP3* and the specific roles of other ingredients in CRD for AD need further experimental verification. Moreover, the specific effects of CRD on microglia and neurons still need to be further studied in in vitro cell experiments.

## Conclusions

In summary, CRD has a neuroprotective effect in 3 × Tg-AD mice. Ferulic acid, rutin, ginsenoside Rg1 and panaxydol may be the main active ingredients of CRD. The therapeutic effect of CRD on AD is achieved through the downregulation of *CASP3* and *EGFR*. The neuroprotective effect of CRD on AD may occur through inhibition of the MAPK pathway to alleviate inflammation. All authors read and approved the final manuscript.

## Supplementary Information

**Additional file 1. Table. S1.** Mass spectrometry parameters of the five detected compounds.

**Additional file 2: Fig. S1.** Total ion chromatograms (TICs) of CRD (**B**) and standard solution (**A**) by ultra-performance liquid chromatography-quadrupole-time-of-flight tandem mass spectrometry (UPLC/Q-TOF-MS).

**Additional file 3: Fig. S2.** Total ion chromatograms (TICs) by ultra-performance liquid chromatography-quadrupole-time-of-flight tandem mass spectrometry (UPLC-Q-TOF-MS). (**A**) TIC of the blank in positive ion mode. (**B**) TIC of brain tissue homogenate of the blank group in positive ion mode. (**C**) TIC of serum of the blank group in positive ion mode. (**D**) TIC of brain tissue homogenate of CRD group in positive ion mode. (**E**) TIC of serum of CRD group in positive ion mode.

**Additional file 4: Fig. S2.** (**F**) TIC of CRD in positive ion mode. (**G**) TIC of the blank in negative ion mode. (**H**) TIC of serum of the blank group in negative ion mode. (**I**) TIC of brain tissue homogenate of the blank group in negative ion mode. (**J**) TIC of serum of CRD group in negative ion mode.

**Additional file 5: Fig. S2.** (**K**) TIC of brain tissue homogenate of CRD group in negative ion mode. (**L**) TIC of CRD in negative ion mode.

**Additional file 6. Table. S2.** Information about ingredients in brain tissue homogenate of blank group.

**Additional file 7. Table. S3.** Information about ingredients in brain tissue homogenate of CRD group.

## Data Availability

The datasets used and/or analyzed during the current study are available from the corresponding author on reasonable request.

## Abbreviations

3 × Tg-AD: Triple-transgenic mouse model of AD
AD: Alzheimer's disease
CRD: Chuanxiong Renshen decoction
CUR: Curtain gas
DS: Discovery Studio 2016
Gas1: Ion Source Gas1
Gas2: Ion Source Gas2
GO: Gene Ontology
IDA: Information-dependent acquisition
IL-17: Interleukin-17
IOD: Cumulative optical density
ISVF: Ion spray voltage floating
KEGG: Kyoto Encyclopedia of Genes and Genomes
LMTM: Leuco-methylthioninium
MT: Methylthioninium
MWM: Morris water maze
PPI: Protein‒protein interaction
TCM: Traditional Chinese medicine
UPLC-Q–TOF–MS: Ultraperformance liquid-chromatography-quadrupole–time-of-flight tandem mass spectrometry
Western blot: Western blot
WT: Wild type
