# Supplementary material for: Integrating network pharmacology, UPLC-Q–TOF–MS and molecular docking to investigate the effect and mechanism of Chuanxiong Renshen decoction against Alzheimer's disease
Source: Chin Med. 2022 Dec 24;17:143. doi: 10.1186/s13020-022-00698-1 (PMC9789652; doi:10.1186/s13020-022-00698-1)
Supplement: Supplementary file 1 — Additional file 1. Table. S1. Mass spectrometry parameters of the five detected compounds. [file 13020_2022_698_MOESM1_ESM.docx]

Mass spectrometry parameters of the five detected compounds.

| Compound ID | Precursor ion | Fragment ion | Accumulation time | Declustering potential | Collision energy | Retention time | Retention time tolerance | Area | Required content(ug/ml) | Actual content(ug/ml) |
| --- | --- | --- | --- | --- | --- | --- | --- | --- | --- | --- |
| Ferulic acid | 415.104 | 267.0662 | 0.1 | -80 | -35 | 5.9 | 30 | 19443 | 1.44 | 2.942 |
| Ginsenoside Rg1 | 193.0508 | 133.0298 | 0.1 | -80 | -35 | 7.9 | 30 | 148000 | 1.944 | 2.926 |
| Puerarin | 845.4901 | 637.4321 | 0.1 | -80 | -35 | 9.75 | 30 | 4701667 | 34.56 | 50.76 |
| Ginkgolide A | 453.1405 | 351.1454 | 0.1 | -80 | -25 | 10.7 | 30 | 128100 | 1.16 | 1.326 |
| Emodin | 269.0463 | 225.0567 | 0.1 | -80 | -35 | 17.35 | 30 | 409233 | 0.72 | 1.397 |
