# Supplementary material for: Integrating network pharmacology, UPLC-Q–TOF–MS and molecular docking to investigate the effect and mechanism of Chuanxiong Renshen decoction against Alzheimer's disease
Source: Chin Med. 2022 Dec 24;17:143. doi: 10.1186/s13020-022-00698-1 (PMC9789652; doi:10.1186/s13020-022-00698-1)
Supplement: Supplementary file 2 — Additional file 2: Fig. S1. Total ion chromatograms (TICs) of CRD (B) and standard solution (A) by ultra-performance liquid chromatography-quadrupole-time-of-flight tandem mass spectrometry (UPLC/Q-TOF-MS). [file 13020_2022_698_MOESM2_ESM.pdf]

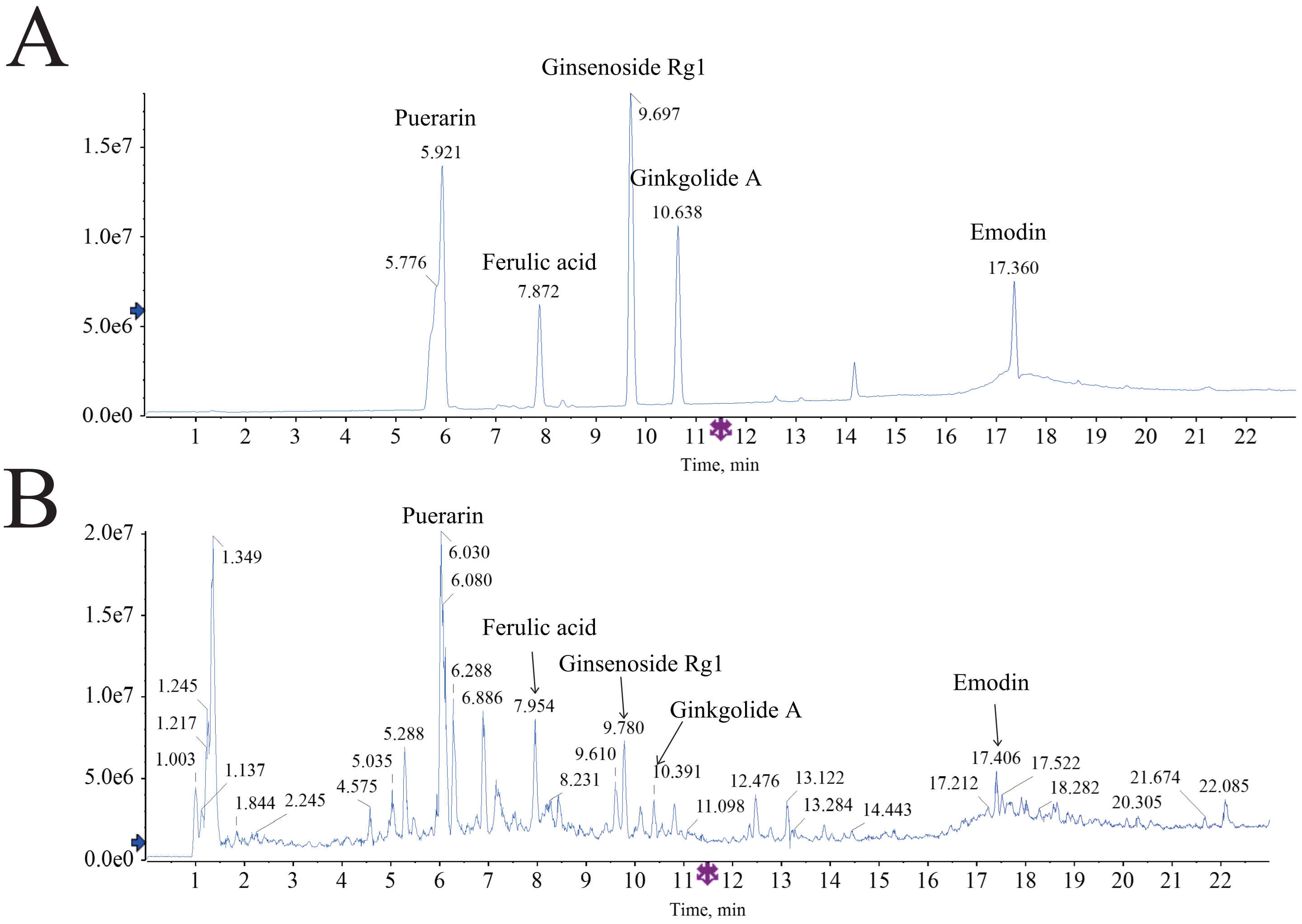

Supplementary Figure 2: The total ion chromatograms total ion chromatogram (TICs) of CRD(B) and Standard solution(A) by ultra-performance liquid chromatography-quadrupole-time-of-flight tandem mass (UPLC/Q-TOF-MS/MS).
