# Supplementary figures and images for: Integrating network pharmacology, UPLC-Q–TOF–MS and molecular docking to investigate the effect and mechanism of Chuanxiong Renshen decoction against Alzheimer's disease
Source: Chin Med. 2022 Dec 24;17:143. doi: 10.1186/s13020-022-00698-1 (PMC9789652; doi:10.1186/s13020-022-00698-1)

A

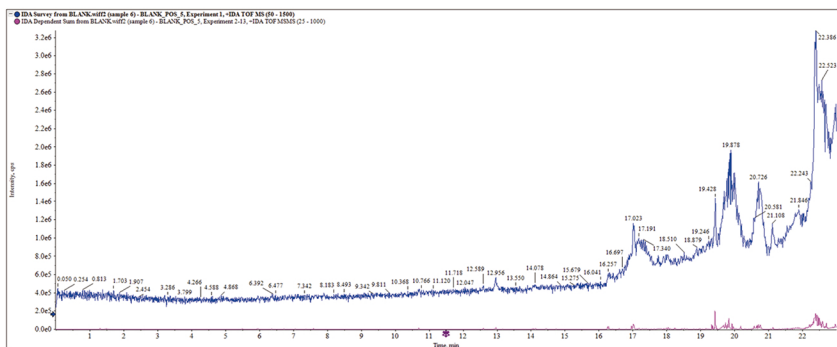

B

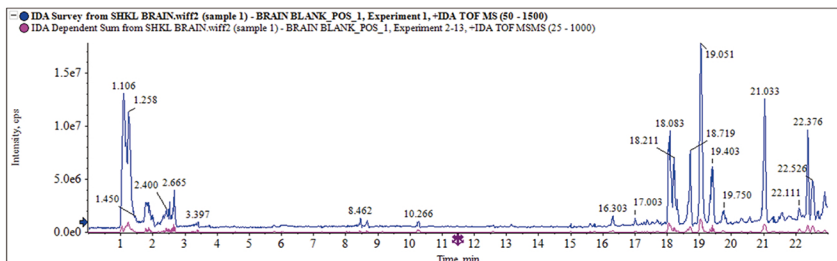

C

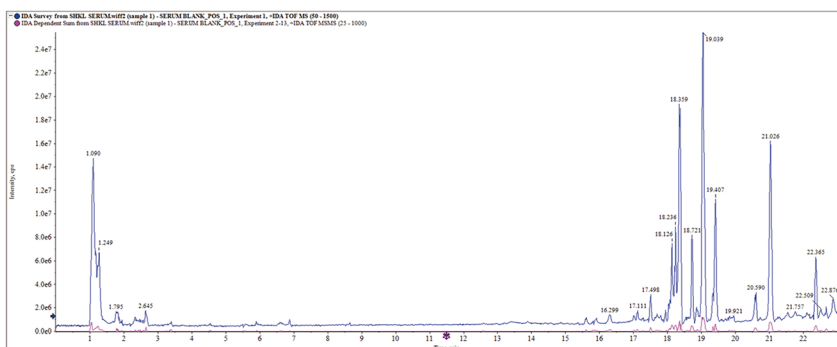

D

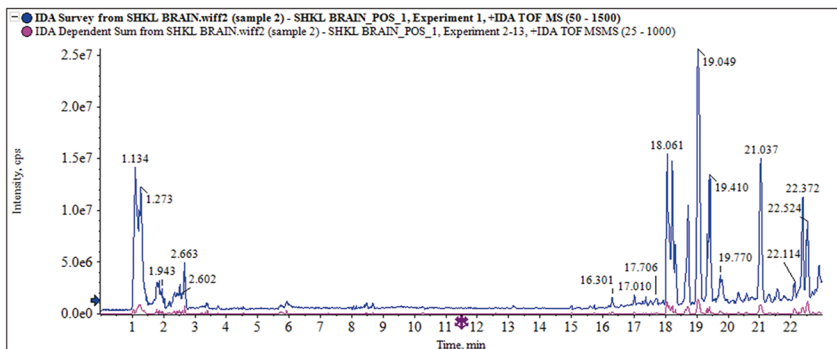

E

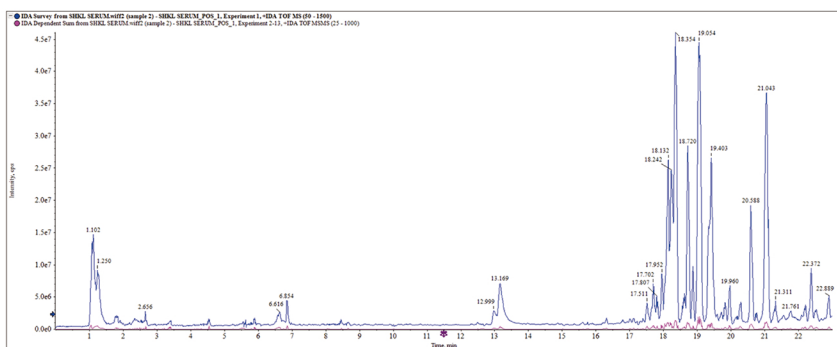

Supplement: Supplementary file 3 — Additional file 3: Fig. S2. Total ion chromatograms (TICs) by ultra-performance liquid chromatography-quadrupole-time-of-flight tandem mass spectrometry (UPLC-Q-TOF-MS). (A) TIC of the blank in positive ion mode. (B) TIC of brain tissue homogenate of the blank group in positive ion mode. (C) TIC of serum of the blank group in positive ion mode. (D) TIC of brain tissue homogenate of CRD group in positive ion mode. (E) TIC of serum of CRD group in positive ion mode. [file 13020_2022_698_MOESM3_ESM.pdf]

F

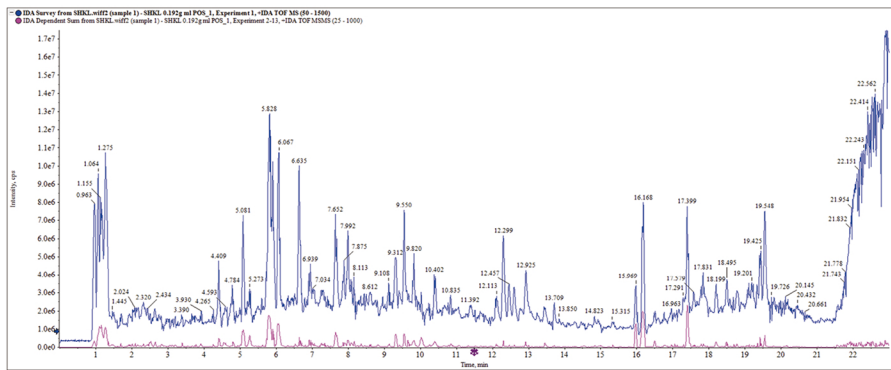

G

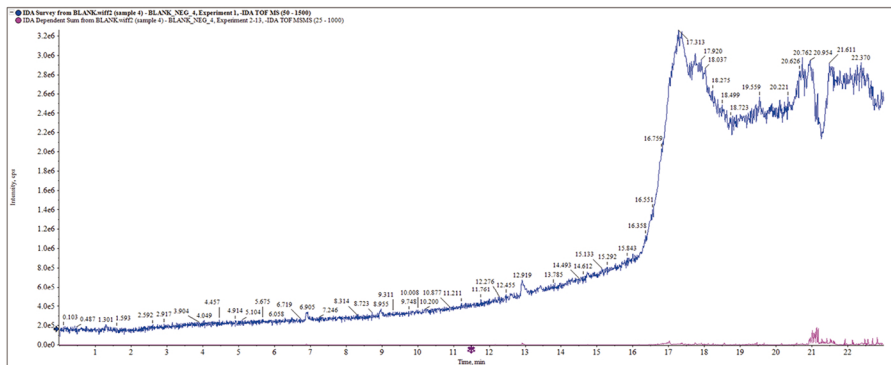

H

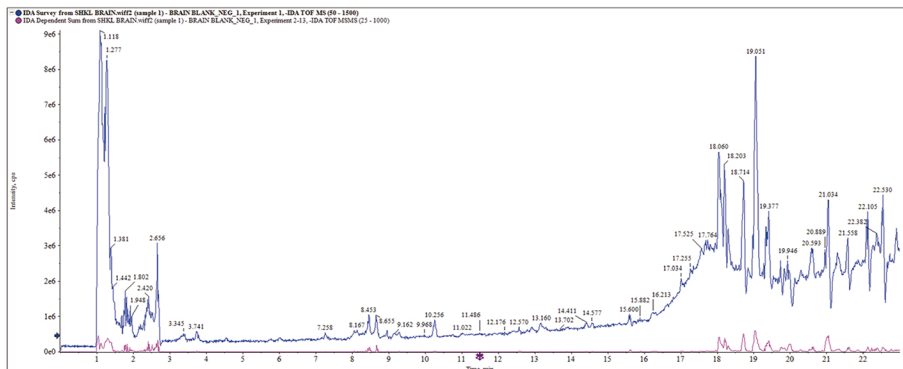

I

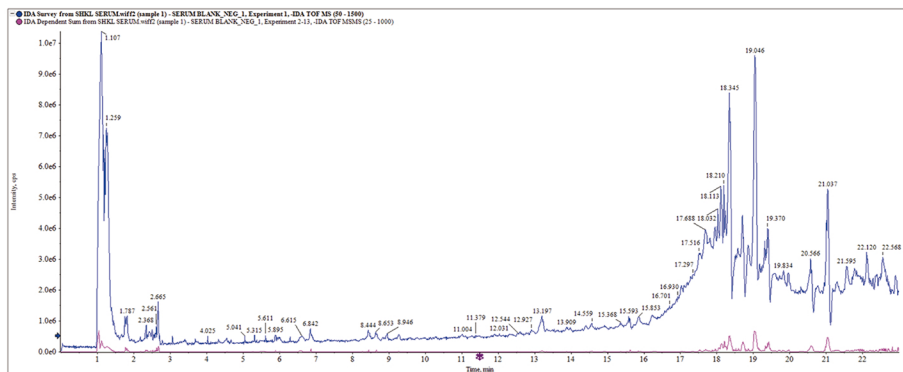

J

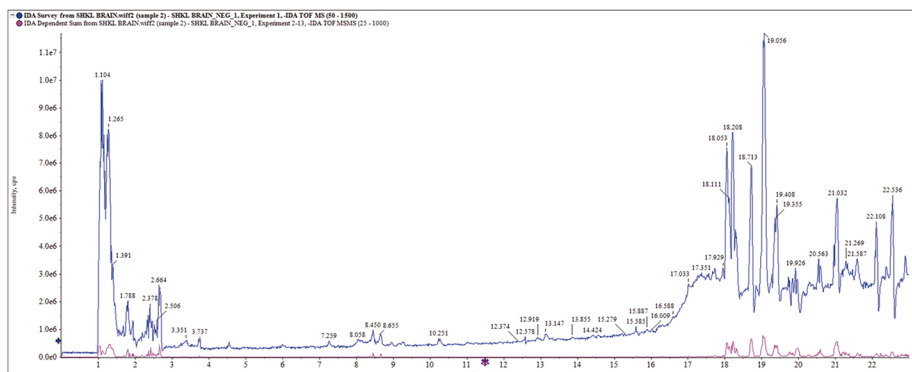

Supplement: Supplementary file 4 — Additional file 4: Fig. S2. (F) TIC of CRD in positive ion mode. (G) TIC of the blank in negative ion mode. (H) TIC of serum of the blank group in negative ion mode. (I) TIC of brain tissue homogenate of the blank group in negative ion mode. (J) TIC of serum of CRD group in negative ion mode. [file 13020_2022_698_MOESM4_ESM.pdf]
