# Supplementary material for: Integrating network pharmacology, UPLC-Q–TOF–MS and molecular docking to investigate the effect and mechanism of Chuanxiong Renshen decoction against Alzheimer's disease
Source: Chin Med. 2022 Dec 24;17:143. doi: 10.1186/s13020-022-00698-1 (PMC9789652; doi:10.1186/s13020-022-00698-1)
Supplement: Supplementary file 5 — Additional file 5: Fig. S2. (K) TIC of brain tissue homogenate of CRD group in negative ion mode. (L) TIC of CRD in negative ion mode. [file 13020_2022_698_MOESM5_ESM.pdf]

K

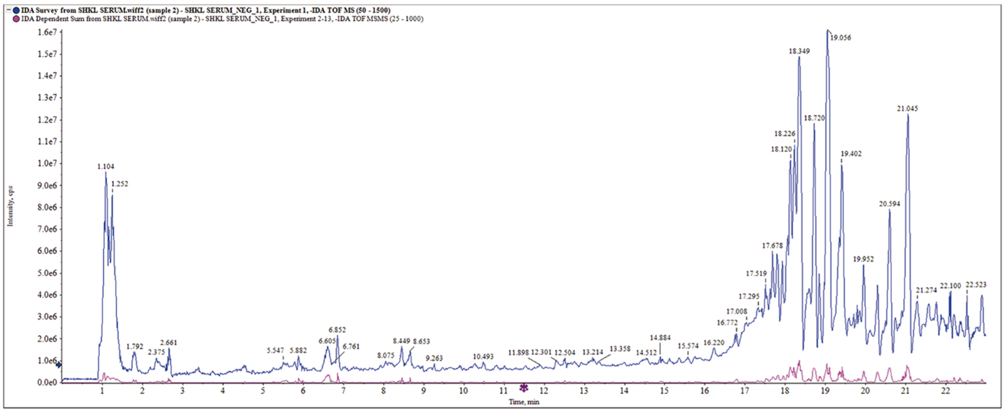

L

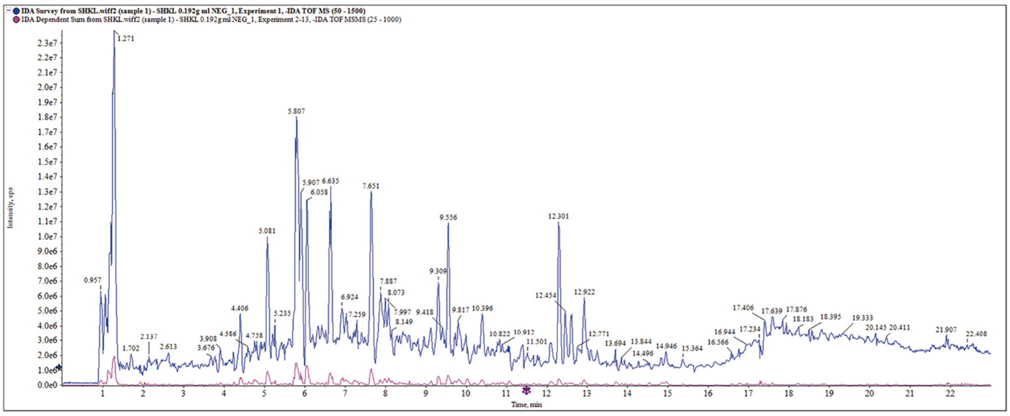

Supplementary Figure 1: The total ion chromatograms total ion chromatogram (TICs) of CRD drug serum and blank serum by ultra-performance liquid chromatography-quadrupole-time-of-flight tandem mass (UPLC/Q-TOF-MS/MS).
(A) TIC of blank in positive ion mode
(B) TIC of brain tissue homogenate of blank group in positive ion mode
(C) TIC of serum of blank group in positive ion mode
(D) TIC of brain tissue homogenate of CRD group in positive ion mode
(E) TIC of serum of CRD group in positive ion mode
(F) TIC of CRD in positive ion mode
(G) TIC of blank in negative ion mode
(H)TIC of blank serum in negative ion mode
(I) TIC of brain tissue homogenate of blank group in negative ion mode
(J) TIC of serum of blank group in negative ion mode
(K) TIC of brain tissue homogenate of CRD group in negative ion mode
(L) TIC of CRD in negative ion mode
