# Supplementary material for: Integrating network pharmacology, UPLC-Q–TOF–MS and molecular docking to investigate the effect and mechanism of Chuanxiong Renshen decoction against Alzheimer's disease
Source: Chin Med. 2022 Dec 24;17:143. doi: 10.1186/s13020-022-00698-1 (PMC9789652; doi:10.1186/s13020-022-00698-1)
Supplement: Supplementary file 6 — Additional file 6. Table. S2. Information about ingredients in brain tissue homogenate of blank group. [file 13020_2022_698_MOESM6_ESM.docx]

**Information about ingredients in brain tissue homogenate of blank group**

| NO | Retention Time | Ingredients Name | Mode | Formula | Mass Error  (ppm) |
| --- | --- | --- | --- | --- | --- |
| 1 | 1.11 | Histidine | POS | C6H9N3O2 | 0.4 |
| 2 | 1.13 | L(+)-Arginine | NEG | C6H14N4O2 | -0.9 |
| 3 | 1.13 | Aspartic acid | POS | C4H7NO4 | 0 |
| 4 | 1.17 | Betaine | NEG | C5H11NO2 | -0.9 |
| 5 | 1.23 | Proline | NEG | C5H9NO2 | -0.4 |
| 6 | 1.73 | Adenine | NEG | C5H5N5 | -0.8 |
| 7 | 1.76 | Cytidine | NEG | C9H13N3O5 | -0.2 |
| 8 | 1.82 | Nicotinic acid | NEG | C6H5NO2 | -0.8 |
| 9 | 1.85 | Nicotinamide | NEG | C6H6N2O | -1 |
| 10 | 2.1 | Citric acid | POS | C6H8O7 | -0.2 |
| 11 | 2.39 | Amber Acid | POS | C4H6O4 | -1 |
| 12 | 2.5 | Adenosine | NEG | C10H13N5O4 | -1 |
| 13 | 2.63 | Guanosine | NEG | C10H13N5O5 | 0.2 |
| 14 | 3.39 | Phenprobamate | POS | C9H11NO2 | -0.2 |
| 15 | 4.55 | L-Tryptophan | POS | C11H12N2O2 | 0.3 |
| 16 | 6.04 | Vitamin B2 | NEG | C17H20N4O6 | -0.1 |
| 17 | 6.96 | Syringaldehyde | NEG | C9H10O4 | -0.1 |
| 18 | 17.84 | Costunolide | NEG | C15H20O2 | -1.3 |
