# Supplementary material for: Integrating network pharmacology, UPLC-Q–TOF–MS and molecular docking to investigate the effect and mechanism of Chuanxiong Renshen decoction against Alzheimer's disease
Source: Chin Med. 2022 Dec 24;17:143. doi: 10.1186/s13020-022-00698-1 (PMC9789652; doi:10.1186/s13020-022-00698-1)
Supplement: Supplementary file 7 — Additional file 7. Table. S3. Information about ingredients in brain tissue homogenate of CRD group. [file 13020_2022_698_MOESM7_ESM.docx]

**Information about ingredients in brain tissue homogenate of CRD group**

| NO | Retention Time | Ingredients Name | Mode | Formula | Mass Error (ppm) |
| --- | --- | --- | --- | --- | --- |
| 1 | 1.12 | Histidine | NEG | C6H9N3O2 | 0.1 |
| 2 | 1.12 | L(+)-Arginine | NEG | C6H14N4O2 | 2 |
| 3 | 1.14 | Aspartic acid | NEG | C4H7NO4 | -0.9 |
| 4 | 1.17 | Betaine | POS | C5H11NO2 | -0.9 |
| 5 | 1.21 | Quinic acid | NEG | C7H12O6 | 1.2 |
| 6 | 1.23 | Proline | POS | C5H9NO2 | -0.6 |
| 7 | 1.61 | Cytidine | NEG | C9H13N3O5 | -4.1 |
| 8 | 1.73 | Adenine | POS | C5H5N5 | -1.5 |
| 9 | 1.86 | Nicotinamide | POS | C6H6N2O | -1.6 |
| 10 | 2.09 | Citric acid | NEG | C6H8O7 | 0.1 |
| 11 | 2.39 | Amber Acid | NEG | C4H6O4 | -2.1 |
| 12 | 2.51 | Adenosine | NEG | C10H13N5O4 | -0.8 |
| 13 | 2.64 | Guanosine | NEG | C10H13N5O5 | -1.8 |
| 14 | 3.39 | Phenprobamate | NEG | C9H11NO2 | -0.2 |
| 15 | 4.55 | L-Tryptophan | NEG | C11H12N2O2 | -0.4 |
| 16 | 6.04 | Vitamin B2 | POS | C17H20N4O6 | 0 |
| 17 | 7.3 | Rutin | NEG | C27H30O16 | -0.6 |
| 18 | 7.69 | Ferulic Acid | POS | C10H10O4 | 1.7 |
| 19 | 9.55 | Ginsenoside Rg1 | NEG | C42H72O14 | -0.5 |
| 20 | 16.79 | Panaxydol | POS | C17H24O2 | -1 |
